# A Systematic Review and Meta-Analysis of Psychological Research on Conspiracy Beliefs: Field Characteristics, Measurement Instruments, and Associations With Personality Traits

**DOI:** 10.3389/fpsyg.2019.00205

**Published:** 2019-02-11

**Authors:** Andreas Goreis, Martin Voracek

**Affiliations:** ^1^Department of Applied Psychology: Health, Development, Enhancement and Intervention, Faculty of Psychology, University of Vienna, Vienna, Austria; ^2^Outpatient Unit for Research, Teaching and Practice, Faculty of Psychology, University of Vienna, Vienna, Austria; ^3^Department of Basic Psychological Research and Research Methods, Faculty of Psychology, University of Vienna, Vienna, Austria

**Keywords:** conspiracy beliefs, conspiracy theories, meta-analysis, systematic review, big five, personality traits, measurement

## Abstract

In the last decade, the number of investigations of the beliefs in conspiracy theories has begun to increase in the fields of social, differential, and experimental psychology. A considerable number of variables have been suggested as predictors of conspiracy beliefs, amongst them personality factors such as low agreeableness (as disagreeableness is associated with suspicion and antagonism) and high openness to experience (due to its positive association to seek out unusual and novel ideas). The association between agreeableness, openness to experience and conspiracy beliefs remains unclear in the literature. The present study reviews the literature of psychological studies investigating conspiracy beliefs. Additionally, the association between Big Five personality factors and conspiracy beliefs is analyzed meta-analytically using random-effects models. Ninety-six studies were identified for the systematic review. A comprehensive account of predictors, consequences, operationalization, questionnaires, and most prominent conspiracy theories is presented. For meta-analysis, 74 effect sizes from 13 studies were extracted. The psychological literature on predictors of conspiracy beliefs can be divided in approaches either with a pathological (e.g., paranoia) or socio-political focus (e.g., perceived powerlessness). Generally, there is a lack of theoretical frameworks in this young area of research. Meta-analysis revealed that agreeableness, openness to experience, and the remaining Big Five personality factors were not significantly associated with conspiracy beliefs if effect sizes are aggregated. Considerable heterogeneity in designs and operationalization characterizes the field. This article provides an overview of instrumentation, study designs, and current state of knowledge in an effort toward advancement and consensus in the study of conspiracy beliefs.

## Introduction

Conspiracy beliefs are usually described as beliefs in the existence of a “vast, insidious, preternaturally effective international conspiratorial network designed to perpetrate acts of most fiendish character” (Hofstadter, 1966, p. 14). The goal of the implied network—the conspiracist actors—is to intentionally deceive and manipulate those involved in, affected by, or witnessing important events, such as war, natural disasters, poverty, and acts of terrorism (Basham, 2003; Stieger et al., 2013). Typically, when official records of an event appear inadequate or no definitive explanation exists for it, conspiracy theories of those events are endorsed (Drinkwater et al., 2012; Dagnall et al., 2015).

A substantial number of people endorse conspiracy theories proposing that the U.S. and U.K. government orchestrated the September 11, 2001 and July 7, 2005 terrorist attacks, respectively (Stempel et al., 2007; Swami et al., 2010, 2011), or that President John F. Kennedy was not assassinated by Lee Harvey Oswald alone (Goertzel, 1994). Because official accounts are insufficient and disbelieved, people are turning to conspiracy theories, defined as “the unnecessary assumption of conspiracy when other explanations are more probable” (Aaronovitch, 2009, p. 5). This disjunction between reality and belief is not necessarily false, considering some contemporary and historical examples where conspiracy did happen—for example, the attempt to experimentally test the effect of mind control by dosing people with LSD in the MKUltra program of the CIA (Wilson and Rose, 2013).

### Current Research

Psychological research has started to generate knowledge about conspiracy beliefs since the mid-1990s (Wood, 2016), but only as recently as the past 10 years, attempts were made to operationalize and measure conspiracy beliefs (Swami et al., 2017; Wood, 2017) and significant ground was made by psychologists in understanding what draws people to conspiracy theories (Lantian et al., 2017). Research has mainly focused on the psychopathological antecedents of conspiracy beliefs, such maladaptive personality traits (e.g., unusual beliefs and experiences, callousness, and eccentricity; Swami et al., 2016c), paranoia (Brotherton and Eser, 2015), and schizotypy (Barron et al., 2014). Considering the relatively high prevalence of conspiracy beliefs, studies also investigated non-pathological individual differences and their associations. Examples are a positive association between conspiracy beliefs and narcissism, self-esteem (Cichocka et al., 2016a), attitudes to authority (Imhoff and Bruder, 2014), social dominance orientation (Swami, 2012), anomia (Wagner-Egger and Bangerter, 2007), and political cynicism (Swami and Furnham, 2012). Lower analytic thinking was also related to conspiracy beliefs (Swami et al., 2014).

Furthermore, the Big Five personality factor openness to experience (intellectual curiosity, active imagination, openness to novel ideas) was positively associated with conspiracy beliefs (Swami et al., 2010, 2011, 2013, 2016b; Orosz et al., 2016). The positive association between openness to experience and conspiracy belief was assumed to be related to the tendency to open individuals to seek novel and unusual ideas and are therefore susceptible to conspiracy (Swami et al., 2013).

Agreeableness was negatively associated, as antagonism and suspicion toward others leads to the endorsement of conspiracy beliefs (Swami et al., 2010, 2013; Swami and Furnham, 2012; Bruder et al., 2013). Additionally, neuroticism and its (sub)pathological elements, such as uncertainty and anxiety, have also been suggested as a predictor (Hollander, 2017). Since reported associations between personality factors and conspiracy beliefs are small yet significant and other studies did not find them (Brotherton et al., 2013; Leiser et al., 2017), exact associations remain unclear.

Conspiracy theories are becoming more popular and the prevalence of some conspiracies are increasing as the actual events they refer to are getting more distant (Goertzel, 1994). Twenty-nine percent of respondents to a survey in 1963 agreed with official accounts of the murder of President Kennedy, yet in 2001, only 13% agreed with official accounts (Carlson, 2001). One study found that conspiracy beliefs are stable over a period of several years (Stieger et al., 2013). Furthermore, people were found to believe in conspiracy theories that contradicted each other, e.g., “Princess Diana faked her own death so that she and Dodi Al-Fayed could retreat into isolation” and “Diana had to be killed because the British government could not accept that the mother of the future king was involved with a Muslim Arab” (Wood et al., 2012, p. 769) were both agreed upon. There are even examples of beliefs in entirely fictitious conspiracy theories (e.g., “The slogan ‘Red Bull gives you wings’ is used because in animal experiments, rats grew rudiment wings,” Swami et al., 2011, p. 455).

### Specific and Generic Conspiracy Beliefs

A key finding of psychological investigations is that measuring the beliefs in specific conspiracy theories is highly related to beliefs in generic ones (Swami et al., 2010). An example item of such generic conspiracy would be “Evidence of alien contact is being concealed from the public” (Brotherton et al., 2013, p. 4), as opposed to the specific “Area 51 in Nevada, US, is a secretive military base that contains hidden alien spacecraft and/or alien bodies” (Swami et al., 2017, p. 14). As both are agreed upon by respondents, this has led to the conceptualization of a stable individual difference variable—called conspiracist ideation—the generalized belief in conspiracy theories (Swami et al., 2011; Brotherton and Eser, 2015).

In his seminal study on conspiracy beliefs, Goertzel (1994) argued that conspiracy beliefs form part of a monological belief system in which conspiratorial ideation serves as evidence for other conspiracist ideation (Swami et al., 2011). In monological belief systems, persons attain explanations for new information that is difficult to explain or that threatens their existing beliefs—new information is therefore simply neglected or inserted in existing belief systems, in this case, they are likely to be of conspiratorial nature (Goertzel, 1994). Incorporated in this are implications for public health and health behaviors (e.g., rejection of vaccination and modern medicine, greater use of alternative medicine; Oliver and Wood, 2014a) as conspiracy beliefs often include pseudoscientific beliefs that reach to the rejection of well-established science (Lobato et al., 2014)

In tandem with this assumption, several questionnaires were created to capture generalized belief in (or ideation of) conspiracies. The Generic Conspiracist Beliefs Scale (GCBS; Brotherton et al., 2013) or the Conspiracy Mentality Questionnaire (CMQ; Bruder et al., 2013), for example, only use items that do not refer to specific conspiracies. An example would be “Groups of scientists manipulate, fabricate, or suppress evidence in order to deceive the public” (Brotherton et al., 2013, p. 8) which taps into aforementioned ideation of such attitudes. In contrast, several other questionnaires exist that tap into endorsement of specific, existing conspiracies like “the moon landings were fake” (e.g., the Conspiracy Theory Belief Scale, CTBS; Douglas and Sutton, 2011; or the Belief in Conspiracy Theories Inventory, BCTI; Swami et al., 2010). Generic scales are, however, less bound by cultural and temporal context than specifically worded questionnaires are and knowledge of specific theories (and therefore, possibly endorsement and/or belief) is likely to be different across cultures and over time (Wood, 2017).

The measurement of conspiracy beliefs and the designs as well as approaches investigate conspiracy beliefs are vast and vary notably between studies. A systematic empirical review of this literature is lacking. Furthermore, there is a substantial literature investigating the relationship of measures of the Big Five personality factors with conspiracy beliefs or indices of conspiracy beliefs with conflicting results. We therefore address this issue by presenting a meta-analysis of all available research examining this relationship and, in particular, testing the hypotheses of the positive association between openness to experience and the negative association of agreeableness and conspiracy beliefs. We additionally provided a descriptive overview of instruments used to measure conspiracy belief, as psychometric properties are described elsewhere (see Swami et al., 2017 for review).

This review provides the first overview of studies measuring conspiracy beliefs in psychological science. It aims to describe (1) the nature and characteristics of psychological research on conspiracy beliefs; (2) the scales, questionnaires, and stimuli used to measure and manipulate conspiracy beliefs; and (3) the nature of associations found between conspiracy beliefs and the variables investigated in the literature. Furthermore, meta-analysis was conducted to analyze the association between Big Five personality factors and conspiracy beliefs, as these associations (especially considering openness to experience and agreeableness) remain unclear in the literature.

## Method

### Inclusion Criteria, Search Strategy, and Data Extraction

**Figure 1:**
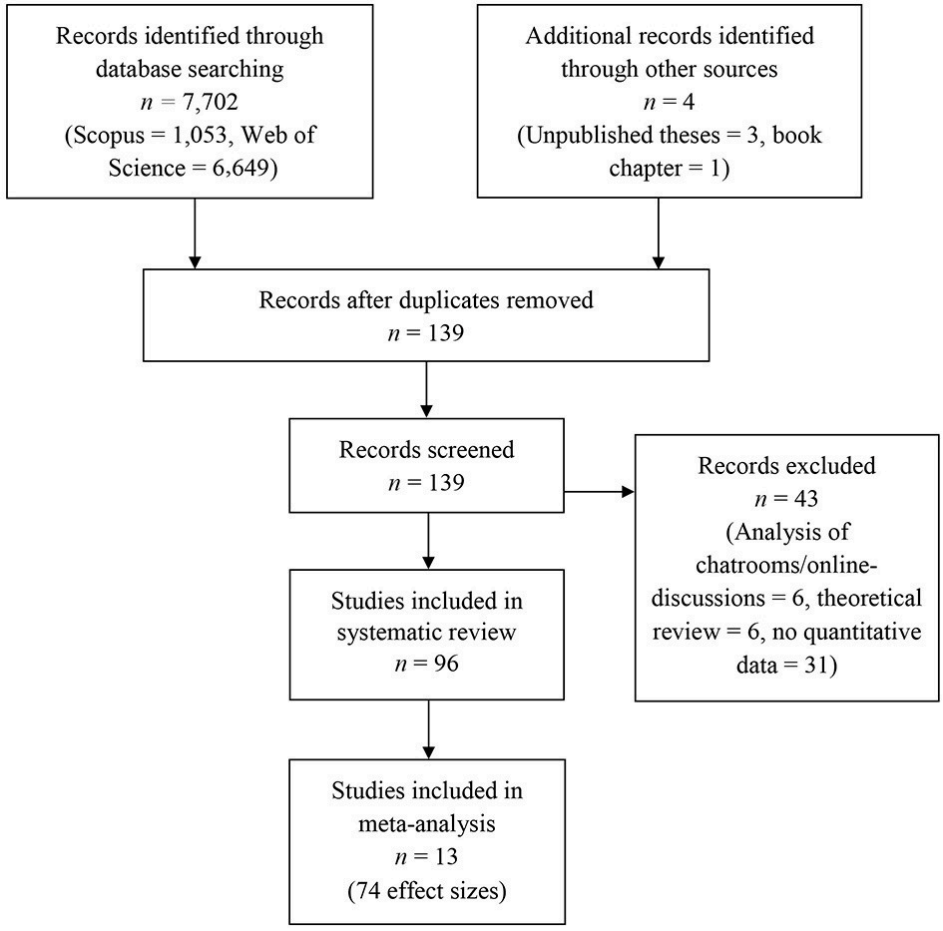
PRISMA flowchart of screening, exclusion, and inclusion criteria.

A search of Scopus and Web of Science was conducted using the keywords “conspir^*^ OR conspira^*^ ideation OR conspira^*^ belief^*^ OR conspira^*^ theory” from the beginning of database-records until March of 2018. Studies were included in this review if they reported empirical, quantitative results of either specific or generic beliefs in conspiracy theories and associations with at least one other variable. No limitations on language or publication status were invoked. In addition, Google Scholar alerts were enabled to ensure inclusion of accepted articles and articles in preprint. The title, abstract, and main text of each paper, thesis, or book (chapter) were examined, with exclusion of documents occurring at each stage (see Figure 1).

The initial search generated 7,702 results. The title and abstracts were screened for eligibility and full text papers were obtained where necessary to evaluate inclusion. After screening, 96 studies (92 peer-reviewed papers, one book chapter, two unpublished dissertations, and one unpublished master's thesis) were identified. Thirteen of the ninety-six studies were included in the meta-analysis, as they reported effect sizes of at least one Big Five personality factor with either the Mini-IPIP (Donnellan et al., 2006), BFI-Short (Rammstedt and John, 2005), TIPI (Gosling et al., 2003), or the abbreviated, 15-item Big Five questionnaire (Furnham et al., 2003). If one of the studies included in the meta-analysis reported associations with more than one questionnaire or item(s), we always included the effect size associated with generic conspiracy beliefs and excluded effect sizes associated with specific conspiracy beliefs (see Abalakina-Paap et al., 1999 for a theoretical discussion of the generic and specific measurement of conspiracy beliefs). This was the case in four studies (Swami et al., 2011, 2013; Swami and Furnham, 2012; Brotherton et al., 2013). We extracted effect sizes (Pearson's *r*) from all studies except for one (Hollander, 2017). In this study, we computed the mean of four correlation coefficients (*r*s = 0.03–0.09) between specific conspiracy beliefs items and neuroticism, resulting in an extracted effect size of *r* = 0.07.

Data of the included studies was entered into a spreadsheet and meta-analysis was conducted with studies reporting effect sizes of Big-Five personality factors and conspiracy beliefs with the package metafor for R (Viechtbauer, 2010). Respective correlation coefficients were transformed to Fisher's *z* and resulting values were then back-transformed into *r* to yield a single measure. Moderator analysis (subgroup analysis for individual conspiracy belief questionnaires and meta-regression for publication year, sample characteristics and reliability of Big Five instrument) was calculated to explain where heterogeneity was high. Egger's regressions were conducted to analyze indications for publication bias. Effect sizes were adjusted using trim-and-fill analyses where indicated. All data and codes are stored and accessible on a repository of the Open Science Framework (doi:10.17605/OSF.IO/HSWB2). A list of all studies included in the systematic review is included in Supplementary Table 1.

## Results

### Study Characteristics

Of the 96 studies, more than 50% were published as recently as between 2015 and 2018 (see Table 1 for details). The literature on conspiracy beliefs has focused extensively on the United States and Europe, as nearly 80% of all studies were conducted on those two continents. Only four studies included exclusively non-western societies (Swami, 2012; Mashuri and Zaduqisti, 2015; Putra et al., 2015; Mashuri et al., 2016). Participants were recruited online (39.8%) or via Amazon's Mechanical Turk (MTurk; 15.7%) with the remaining 44.6% being recruited in offline, face-to-face settings. More than 45% of all samples consisted of adults from the general population, not including the 15.7% of studies using MTurk workers as a sample, which almost exclusively were general population samples. The remainder of samples comprise entirely of (under)graduates. Two studies included in the review used data from representative samples in the United States (Hollander, 2017) and Italy, respectively (Mancosu et al., 2017). Furthermore, more than 70% of all studies used a cross-sectional study design (see Table 1 for detailed sample characteristics).

**Table 1:** Characteristics of the 96 studies included in this review.

**Characteristic**	**No. of studies**	**% of total studies**
**YEAR OF PUBLICATION**		
1994–2000	2	2.1
2001–2007	2	2.1
2008–2014	36	37.5
2015–2018	56	58.3
**JOURNAL OF PUBLICATION**		
Personality and individual differences	10	10.4
Frontiers in psychology	8	8.3
Applied cognitive psychology	7	7.3
British journal of psychology	5	5.2
PLoS ONE	6	6.3
European journal of social psychology	4	4.2
Remaining journals (each with < 4 published studies)^a^	56	58.3
**CONTINENT OF ORIGIN**		
Europe	42	43.8
USA	34	35.4
Asia	4	4.2
Australia/New Zealand	3	3.1
Africa	0	0
Multiple continents	13	13.5
SAMPLE SIZE^*^		
0–100	27	16.3
101–200	46	27.7
201–300	35	21.1
301–400	16	9.6
401–500	9	5.4
>500	33	19.9
SAMPLE^*^		
Adults	79	47.6
Undergraduates	22	13.3
Students	39	23.5
MTurk workers	26	15.7
METHOD OF DATA COLLECTION^*^		
Face-to-face	74	44.6
Online	66	39.8
MTurk	26	15.7
STUDY DESIGN^*^		
Cross-sectional	118	71.1
Experimental manipulation	48	28.9

*Total number of publications = 96. Total number studies including multi-study publications = 166*.

* *Counts including multi-study publications*.

a *Includes three unpublished theses and one book chapter*.

### Questionnaires

Thirteen questionnaires have been used to measure conspiracy beliefs in the included studies; six of them measure a generic form of conspiracy beliefs (see Table 2). Two of most-used scales (GCBS; Brotherton et al., 2013; and CMQ; Bruder et al., 2013) measured generic beliefs without items about specific (known or unknown) conspiracies. A multitude of studies used single- or multi-item measures for specific conspiracy theories that were not categorized as a defined scale or questionnaire. Specific items range from local conspiracies (e.g., “City hall corruption during the construction of a local metro line in Amsterdam,” van Prooijen and Acker, 2015, p. 755) to entirely made up ones (e.g., “Smoke detectors emanate dangerous hyper sound,” Imhoff and Lamberty, 2017, p. 643).

**Table 2:** Questionnaires used in the studies included in the review.

**Questionnaire**	**Acronym**	**Original study**	**Generic form of beliefs?**	**Used in studies^a^**
Generic Conspiracist Beliefs Scale	GCBS	Brotherton et al., 2013	Yes	33
Belief in Conspiracy Theories Inventory	BCTI	Swami et al., 2010	No	27
Conspiracy Mentality Questionnaire	CMQ	Bruder et al., 2013	Yes	22
Conspiracy Theory Belief Scale	CTBS	Douglas and Sutton, 2011	No	8
Generalized Conspiracy Belief Scale	–	Rose, 2017	Yes	6
Specific Conspiracy Belief Scale	–	Rose, 2017	No	6
Conspiracy Theory Questionnaire	CTQ	Darwin et al., 2011	Yes	5
One-Item Conspiracy Measure	OICM	Lantian et al., 2016	Yes	5
Flexible Inventory of Conspiracy Suspicions	FICS	Wood, 2017	No	3
General Measure of Conspiracism	GMC	Drinkwater et al., 2012	Yes	3
Conspiracy Beliefs Scale^b^	CBS	Kumareswaran, 2014	No	2
Endorsement of Specific Conspiracy Theories	ESCT	Irwin et al., 2015	No	1
Belief in Commercial Conspiracy Theories Inventory	–	Furnham, 2013	No	1

a *Including multi-study papers*.

b *Currently unpublished*.

Several terror-related conspiracy items doubting official accounts of the 9/11 (Swami et al., 2010; Brotherton et al., 2013; Carey et al., 2016; Moulding et al., 2016) or 7/7 terror attacks (e.g., Swami et al., 2011, 2014; Brotherton et al., 2013) and about the perpetrators (e.g., Osama bin Laden; Wood et al., 2012) have also been used throughout the literature. Furthermore, recent events with extensive media coverage, such as the disappearance of Malaysian Airlines 777 in 2014 (Marchlewska et al., 2017), the abduction and subsequent captivation of Natascha Kampusch in Austria (Stieger et al., 2013), and the “Deflategate” controversy (Tom Brady, a New England Patriots quarterback conspired to reduce air pressure in footballs used during a playoff game; Carey et al., 2016). Commercial conspiracies, considering subliminal advertising and marketing tricks were investigated as well (Furnham, 2013).

Beliefs in contemporary conspiracy theories, such as those doubting the scientific facts about anthropogenic climate change (Lewandowsky et al., 2013a,b; Jolley and Douglas, 2014b; Van der Linden, 2015; van Prooijen et al., 2015) and medical conspiracies (Oliver and Wood, 2014a; Pavlova and Silbereisen, 2015; Galliford and Furnham, 2017; Lahrach and Furnham, 2017) were used extensively. Amongst them are the beliefs that pharmaceutical companies cover up harmful side effects to continuously make profits and the rejection between the link of smoking and cancer (Jolley and Douglas, 2014a; Wood, 2017). Another example of a widespread assumption is the belief that vaccinations cause autism in children (Jolley and Douglas, 2017), dating back to a fabricated (and subsequently retracted) scientific paper in The Lancet (Jolley and Douglas, 2017).

Conspiracies concern members of an outgroup range from those against Jews (Swami, 2012; Grzesiak-Feldman, 2013) to Islamophobia (Imhoff and Bruder, 2014; Mashuri and Zaduqisti, 2015; Swami et al., 2018) to specific groups of nationalities, both immigrants or living in their homeland (e.g., anti-Russian and anti-Polish, Cichocka et al., 2016b). Studies conducted in eastern European countries investigated conspiracy beliefs about the European Union, their involvement in deliberately attracting and letting refugees into eastern European countries and, more generally, plotting secret actions against those countries to destroy their economy and culture (Marchlewska et al., 2017). However, similar studies were performed in Germany (Uenal, 2016) and the United Kingdom (Swami et al., 2018).

### Predictors of Conspiracy Beliefs

A detailed overview of all variables predicting beliefs in conspiracies used in the studies included in this review is depicted in Supplementary Table 1. A multitude of studies view conspiracy beliefs as a symptom of an underlying psychological disorder, the prodromal phases of a psychological disorder or the traits associated with them. Amongst those, paranoia (Bruder et al., 2013), paranoid ideation, and schizotypy (Darwin et al., 2011) were prominently found to harbor connections with conspiracy beliefs. Paranoid ideation and schizotypy share similar traits, including suspicion, magical thinking, and odd and unusual beliefs (Barlow and Durand, 2009). In paranoid ideation, people are harboring thoughts that external agents have an intention of hostility toward them; this hostility may be in the form of physical or verbal threats and, relevant for conspiracy beliefs, fearing deception, exploitation, and disloyalty (Freeman et al., 2005; Darwin et al., 2011).

#### Personality Traits

Fear and anxiety were reported as positive predictors of conspiracy beliefs (e.g., Grzesiak-Feldman, 2013). As people are anxious, fear a threatening situation, or have low perceived feelings of control over situations, they tend to conspiracies. Both state and trait anxiety are positive predictors of conspiracy beliefs (Grzesiak-Feldman, 2013; Swami et al., 2016a). The need to exert control over one's social environment, operationalized as feelings of control (Leiser et al., 2017; van Prooijen, 2017), desirability of control (Lobato et al., 2014; Rose, 2017) were all stable predictors of conspiracy beliefs. Coupled with the desire to have perceived control over the environment is the general concept of making sense of the world. Such sense making-motivation is central for conspiracy theories, as it provides explanations for events and, most of the time, an entity to blame (van Prooijen and van Dijk, 2014).

Paranormal belief, a construct related to paranoid ideation and schizotypy, operationalized as the acceptance of processes and phenomena that are scientifically impossible (e.g., precognition, psychokinesis, extra-sensory perception) was positively linked to conspiracy beliefs as well (Darwin et al., 2011). Paranormal belief also includes magical, superstitious, and religious thinking (Lindeman and Aarnio, 2006). People with pronounced paranormal belief doubt orthodoxies and scientific knowledge (Ramsay, 2006), and this led to the assumption that if orthodoxies are doubted in one area (such as the belief in ghosts); they are doubted in other areas (such as official explanations of events or catastrophes) as well, thus explaining the path to conspiracy beliefs. The proneness to statistical errors and failures in probabilistic reasoning is a key element of paranormal belief (Dagnall et al., 2007). This holds true for the belief in conspiracy theories as well, as participants with susceptibility to conjunction fallacy errors were more likely to believe conspiracies (Brotherton and French, 2014; Dagnall et al., 2017). The same is true for illusory correlations, confirmation bias, and hindsight bias (Shermer, 2010, 2011). Conspiracy beliefs may be, at least in part, a product of bias or shortcuts made when searching for explanations (Brotherton and French, 2014).

The role of self-evaluation was also found to be an important link, people with a high amount of narcissism, an exaggerated feeling of self-love, were more prone to believe in conspiracies (Kumareswaran, 2014; Cichocka et al., 2016a). Narcissism is positively associated with paranoid thinking, as narcissists are perceiving the actions of others intentionally targeted against themselves (Fenigstein and Vanable, 1992). Such perceptions, again, are linked to conspiracy beliefs. Self-esteem, the positive self-evaluation without narcissistic components (Paulhus et al., 2004), seems to be negatively associated with conspiracy beliefs. Conspiracies are appealing to people who lack confidence and excess self-promotional characteristics, such as self-esteem (Cichocka et al., 2016a; Galliford and Furnham, 2017).

#### Social and Political Factors

A second branch of the literature argues that investigating only clinical or subclinical correlates of conspiracy beliefs is insufficient. These studies tried to connect socio-political, value related, and religious attitudes to conspiracy beliefs. Given the high prevalence of the endorsement of conspiracy theories, a disorder-centered view could never fully explain the support and acceptance of them (Oliver and Wood, 2014b; Mancosu et al., 2017). Socio-political variables, e.g., political cynicism (Swami et al., 2010; Swami, 2012) and negative attitudes toward authority (Swami et al., 2011; Cichocka et al., 2016b), were investigated. Anomia, the concept that describes the perception that the complexity of modern societies has become unintelligent (Goertzel, 1994; Bruder et al., 2013), was positively associated as well. A high level of anomia is an indicator that a person feels alienation and disaffection from societal systems (Goertzel, 1994) and thus endorses conspiracies—blaming an external agent for their low social-political power. The same association is true for persons who feel alienated because of unemployment or perceived status of their in-group (Uenal, 2016). Ethnic minority status was associated as well, as members of minority groups reported stronger beliefs (Wilson and Rose, 2013).

Religious individuals are more likely than non-religious to believe in conspiracy theories (Oliver and Wood, 2014b; Lahrach and Furnham, 2017). Political attitudes, orientation, or affiliation—an item presented to participants in over 40 studies included in this review—revealed a quadratic association at either side of the political spectrum (Swami and Furnham, 2012; Pasek et al., 2015; van Prooijen et al., 2015; Lahrach and Furnham, 2017; Mancosu et al., 2017). Political extremism, either to the left or to the right, is associated as an attempt or thinking style, aimed at, once again, making sense of societal events (van Prooijen et al., 2015). Additionally, right-wing authoritarianism was a positive predictor—a political attitude characterized by obedience to an authoritarian leader, a deeply rooted mentality when it comes to traditional societal values, and, at the same time, a distrust against governmental structures (Imhoff and Bruder, 2014; Richey, 2017).

Closely related to right-wing authoritarianism is social-dominance orientation, a measure of preference for hierarchical social system (Swami, 2012). Imhoff and Bruder (2014) argued that persons with high amounts right-wing authoritarianism endorse conspiracies that involve deviant, high-power groups (e.g., anti-Sematic conspiracies), that threaten the status quo. Social dominance-orientation, on the other hand, leads to an endorsement of theories involving the deviance of low-status groups (e.g., homosexuals, ethnic minorities), as they are thought to threaten the status quo as well. Conspiracy beliefs, right-wing authoritarianism and social dominance orientation operate as system-justifying functions, as a defense system to protect the socio-political status quo (Goertzel, 1994; Imhoff and Bruder, 2014).

Several studies have stressed the negative relationship between scientific knowledge, rational thinking and conspiracy beliefs. People who are more used to analytic thinking are not as prone to fall for the logical fallacies inherited in conspiracy theories (Wagner-Egger and Bangerter, 2007; Swami et al., 2014; Ballová Mikušková, 2017). Lower intelligence was also associated with conspiracy beliefs (Stieger et al., 2013; Ballová Mikušková, 2017). One study presented participants with analytic-thinking prime (a scrambled, hard to read font); resulting in a decrease of conspiracy belief scores (Swami et al., 2014). In general, people with high education are less likely than people with low education to believe in conspiracy theories (van Prooijen, 2017).

### Association With Big Five Personality Factors

**Figure 2:**
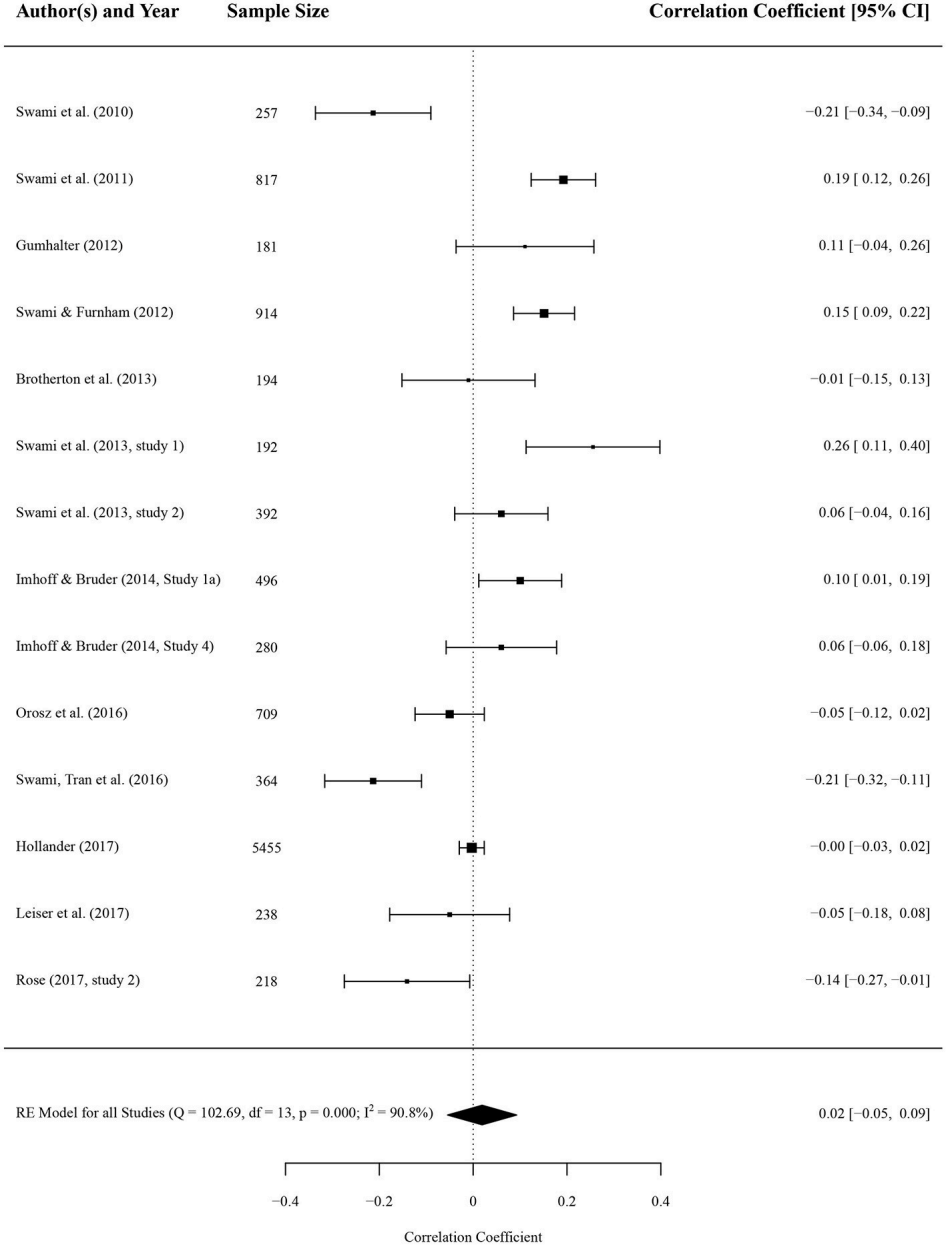
Forest plot of correlation coefficients between conspiracy beliefs and openness to experience. A positive effect size indicates that higher levels of conspiracy beliefs is associated with higher levels of openness to experience. Average effect was calculated using a random-effects model.

**Figure 3:**
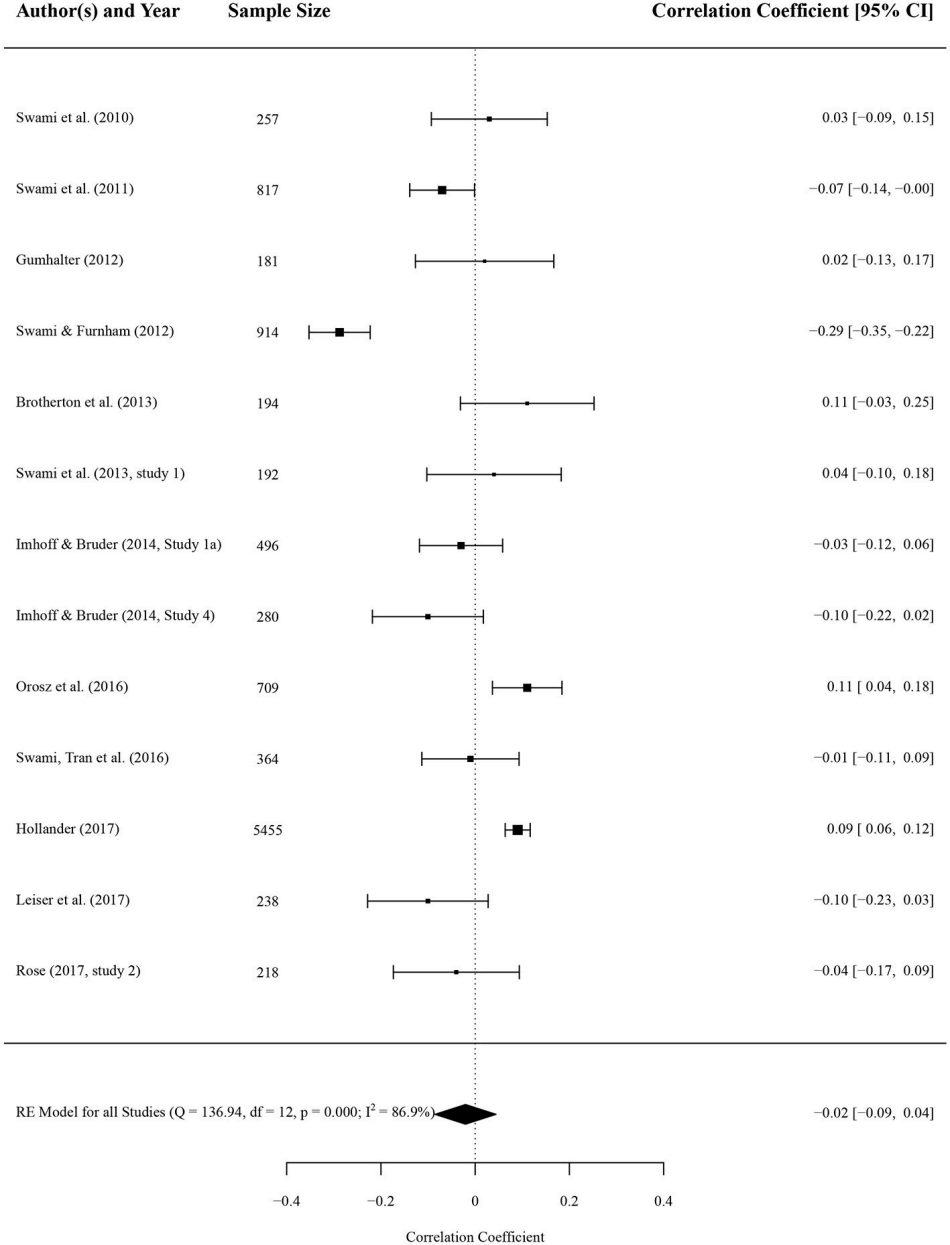
Forest plot of correlation coefficients between conspiracy beliefs and agreeableness. A positive effect size indicates that higher levels of conspiracy beliefs is associated with higher levels of agreeableness. Average effect was calculated using a random-effects model.

Results of the meta-analysis concluded that none of the Big Five scales had an effect on conspiracy beliefs if effect sizes are combined. Forest plots of the results for openness to experience and agreeableness are depicted in Figures 2, 3, respectively. Forest plots of the remaining three Big Five personality factors (neuroticism, conscientiousness, and extraversion) are depicted in the Supplementary Material. There were 74 effect sizes extracted from 13 studies covering data from 12,086 persons, 14 effect sizes for openness to experience (*n* = 5,252), 13 effect sizes for agreeableness (*n* = 10,315), 15 effect sizes for conscientiousness (*n* = 11,001), and 16 effect sizes for extraversion (*n* = 6,001) and neuroticism (*n* = 11,456). With openness to experience, a correlation coefficient of *r* = 0.02 (*p* = 0.612) with a 95% confidence interval (CI) of −0.06 to 0.09 emerged. Similar results were found for agreeableness (*r* = −0.02, 95% CI −0.09 to 0.04, *p* = 0.534), conscientiousness (*r* = 0.01, 95% CI −0.02 to 0.05, *p* = 0.433), extraversion (*r* = 0.01, 95% CI −0.02 to 0.03, *p* = 0.575), and neuroticism (*r* = 0.03, 95% CI −0.02 to 0.09, *p* = 0.204). None of the overall associations were significant (all *p*s > 0.05).

The variance across studies for openness to experience was *I*^2^ = 90.75 [*Q*_(13)_ = 102.69, *p* = < 0.001], similarly high heterogeneity emerged for agreeableness [*I*^2^ = 86.86, *Q*_(12)_ = 136.94, *p* < 0.001], consciousness [*I*^2^ = 57.20, *Q*_(14)_ = 35.53, *p* = 0.001], and neuroticism [*I*^2^ = 82.52, *Q*_(15)_ = 78.88, *p* < 0.001]. Only the variance across studies investigating effects of extraversion on conspiracy beliefs was comparably low [*I*^2^ = 18.65, *Q*_(15)_ = 20.43, *p* = 0.156].

#### Moderator Analysis

We computed subgroup-analyses (by inventory, BCTI against all other inventories; and by form of conspiracy beliefs, generic against specific) for openness to experience and agreeableness due to high heterogeneity and because of the attention it received in the literature on conspiracy beliefs. With openness to experience, six studies used the BCTI and two studies the CMQ, the remaining studies all used different questionnaires or single items. Five studies measured generic and 9 specific conspiracy beliefs. There were no difference between questionnaires used [*r* = −0.04 and *r* = 0.09, *Q*_(1)_ = 3.01, *p* = 0.083] and between form of conspiracy beliefs [*r* = −0.01 and *r* = 0.03, *Q*_(1)_ = 0.27, *p* = 0.601]. For agreeableness, five studies used the BCTI (eight studies used others); five studies measured generic and eight measured specific conspiracy beliefs. Again, there was no difference between questionnaires [*r* = −0.06 and *r* = 0.01, *Q*_(1)_ = 1.07, *p* = 0.301] and form of conspiracy beliefs [*r* = 0.01 and *r* = −0.04, *Q*_(1)_ = 0.67, *p* = 0.412].

Multiple meta-regression for the association with openness to experience showed a significant effect (with a negative coefficient) for the proportion of women in the sample, indicating that samples with more males reported stronger positive associations (see Table 3 for full list of coefficients). Furthermore, younger samples and studies outside of Europe were positive predictors of higher associations of openness and conspiracy beliefs. Reliability of the Big Five instrument used was negatively associated with the strength of the association. Our meta-regression explained 29.12% of the heterogeneity, indicating that a non-trivial proportion of overall heterogeneity (*I*^2^ was originally 90.75 for openness) remains unexplained. For the meta-regression on the association with agreeableness, only age was significant with a positive coefficient. Older samples reported higher associations of conspiracy beliefs and agreeableness. Our meta-regression model explained 26.17% of heterogeneity, again, a small proportion of the original *I*^2^ = 86.86, rendering non-trivial effects of unexplained heterogeneity for agreeableness (see Table 3 for full list of coefficients). Method of data collection (paper-pencil, online, or MTurk) did not significantly predict associations for both openness and agreeableness.

**Table 3:** Parameters of mixed-effects meta regression on associations of openness to experience, agreeableness, and conspiracy beliefs.

**Predictors**	**Openness to experience (*****k*** **=** **14)**			**Agreeableness (*****k*** **=** **13)**		
	***b***	***SE***	***p***	***b***	***SE***	***p***
Year of publication	0.028	0.027	0.288	0.004	0.020	0.824
Percentage of women in sample	−0.055	0.021	**0.008**	0.011	0.014	0.455
Mean age of sample	−0.012	0.005	**0.025**	0.011	0.005	**0.033**
PP vs. online	−0.192	0.174	0.269	0.141	0.190	0.457
PP vs. MTurk	0.339	0.175	0.053	−0.005	0.091	0.955
Reliability of the big five instrument	−1.828	0.753	**0.015**	0.455	0.408	0.265
Europe vs. other continent	−0.502	0.217	**0.021**	0.055	0.122	0.650
***R***^**2**^ (*Q*)	**29.12** (10.911)		0.143	**26.17** (9.302)		0.232

*k, number of samples; b, unstandardized regression coefficient; SE, standard error of unstandardized coefficient; PP, paper-pencil. p-values in boldface denote significant coefficients (p < 0.05)*.

#### Dissemination Bias

Egger's regressions to test for funnel plot asymmetry indicated publication bias for the summary effect of conscientiousness (*z* = −2.65, *p* = 0.008). No indication for publication bias was found in the reaming four personality factors (*p*s 0.371–0.782). Trim and fill analysis for conscientiousness resulted in six studies missing on the right side of the funnel plot and the recomputed summary effect was *r* = 0.05 (CI 0.01–0.09, *p* = 0.009), indicating a significant small (yet negligible) effect if missing studies were accounted for.

## Discussion

The present work reviewed existing the psychological literature about conspiracy beliefs. In the 96 studies included in this review, a wide range of variables were investigated and associated with conspiracy beliefs. Furthermore, a multitude of questionnaires and single-items are available and readily used in the literature about conspiracy beliefs. The field itself is a particular young one—building on Goertzel's (1994) seminal work, only three studies (McHoskey, 1995; Stempel et al., 2007; Wagner-Egger and Bangerter, 2007) fulfilling the inclusion criteria were published between 1994 and 2007. The remaining 93 studies were published after 2007. The same applies to every questionnaire measuring conspiracy beliefs.

Thirteen questionnaires used to operationalize and measure conspiracy beliefs were identified. Other authors (Swami et al., 2017) have previously noted that psychometric properties going beyond reliability are hardly ever examined, with factorial as well as convergent validity remaining unknown, thus raising concern of the bias in studies and amount of noise measured (see Swami et al., 2017 for a detailed review of psychometric properties). Overall, only three studies subjected their data to factor analysis (Swami et al., 2010; Wood et al., 2012; Rose, 2017) and this number did not increase in the studies reviewed here. In their review of psychometric properties, Swami et al. (2017) recommended the use of the GCBS and the CMQ. The One-Item Conspiracy Measure (OICM; Lantian et al., 2016) should not be used in future studies due to poor factorial and construct validity. It is essential for future studies to validate measurements of conspiracy beliefs or, at least, report factorial validity.

No significant effects were found in the meta-analysis of the association of between Big Five personality factors and conspiracy beliefs. Low to medium negative effects of agreeableness (Swami et al., 2011; Swami and Furnham, 2012) and positive effect of openness to experience (Swami and Furnham, 2012; Swami et al., 2013) were reported and subsequently cited throughout the literature. The current work adds to the literature that associations between openness to new experience and agreeableness do not exist if effect sizes are combined. Our study shows that these associations could not be explained by the questionnaire used or by the form of conspiracy beliefs measured (i.e., generic of specific conspiracy beliefs). The remaining Big Five personality factors did not gain as much attention as agreeableness and openness to experience, only neuroticism was seldom referred as a possible predictor (Hollander, 2017), because of its inclusion of frustration and hopelessness. Studies reporting null findings for the effects of agreeableness and openness (Orosz et al., 2016; Hollander, 2017) are relatively new and provide larger sample sizes, explaining why significant effects were acknowledged until recently. Future research should avoid theoretical assumptions about the associations of classical personality variables, such as the Big Five, as they are null findings.

Potentially negative consequence of the endorsement of conspiracy theories are numerous. Feeling powerless leads to a potential disengagement in politics and society (Jolley and Douglas, 2014b). Furthermore, health behaviors are reduced, the prevention of pregnancy and sexually transmitted diseases is affected (Bogart and Thorburn, 2006), and, additionally, the intention to get vaccinations or receive medical treatments (Oliver and Wood, 2014a). However, positive effects might also be explored in the future. If the tendency to pathologize individuals with conspiracy beliefs is put aside (yet not disputed), it may be viewed as a tool for people to challenge social hierarchies and encourage government transparency (Jolley and Douglas, 2014b), creating a more balanced and nuanced conceptualization.

The reasons why people endorse or believe in conspiracy theories are diverse. Reported individual effects are mostly small and treated in isolation. Conspiracies appear to appeal to those who feel disconnected from society, who are unhappy or dissatisfied with their circumstances, who possess a subjective worldview that includes unusual beliefs, experiences and thoughts, and do not feel in control of their life (c.f. Rose, 2017). The endorsement of them challenges existing power structures in society (Imhoff and Bruder, 2014). Furthermore, those with higher levels of clinically relevant traits such as paranoid thought and schizotypy endorse them. Conceptually, for a comprehensive understanding of the causes of conspiracy beliefs, interaction of predictors should be investigated. Anomia, for example, combines elements of social-dominance orientation, powerlessness, need for cognitive closure, and distrust (Lamberty et al., 2018), yet variables are often treated independently.

Furthermore, the literature on conspiracy beliefs lacks a theoretical framework. Some authors have studied conspiracy beliefs within the dual-process motivational model of intergroup attitudes and prejudice (Wilson and Rose, 2013); identifying and testing paths leading to a conspiratorial worldview, yet most of the studies included were not based on any theoretical framework. The assumption of an underlying monological beliefs system was also questioned, as conspiracy theories are not always mutually supportive, and the assumption itself lacks empirical evidence (Sutton and Douglas, 2014). Developing, examining, and testing theoretical frameworks as well as the evaluation of a valid operationalization of conspiracy beliefs is therefore crucial. Given that conspiracy theories influence our attitudes more than we realize (Douglas and Sutton, 2008), future investigations to understand more about the psychology of conspiracy beliefs are vital.

The meta-analyses in the present study is based on correlations between Big Five personality factors and conspiracy beliefs only. Third variables, which are not possible to control for, might have influence the extracted associations between the personality factors. Additionally, a multitude of questionnaires or single-items was used in the literature to measure and operationalize them. We tried to account for this high amount of heterogeneity by analyzing subgroups and meta-regression where possible. Neither the questionnaires used to measure conspiracy beliefs nor the form of conspiracy beliefs (generic or specific) explained a significant amount of heterogeneity in the present study. Significant moderators in meta-regression such as age, gender, and geographic location of samples revealed bias in associations, but did not reduce heterogeneity to non-trivial amounts. Crucially, reliability of Big Five instruments negatively predicted the association of openness to experience and conspiracy beliefs, implying measurement bias that could have led to the assumption of significant associations in the current literature, as most studies used short or abbreviated (yet validate) forms of Big Five instruments. Most studies included in the review used cross-sectional designs; however, only a third of samples used student or undergraduate samples. Coding of study characteristics was performed only by one author (AG) due to nature of low inference codes necessary for this meta-analysis (Card, 2012).

This first systematic review provides an overview of the current state of knowledge, study designs, instruments, and operationalization of studies investigating conspiracy beliefs. In conclusion, the field is marked by a high heterogeneity of instrumentation and designs. We furthermore provided evidence that none of the Big Five personality dimensions are associated with conspiracy beliefs. Our review of the literature demonstrated that personality as such is clearly associated with conspiracy beliefs, yet these associations are not readily captured with inventories measuring the Big Five. A compressive overview of instruments available to scholars is provided and discussed. This review provides a first effort to advance the field of psychological investigations of conspiracy beliefs toward consensus and advancement.

## Author Contributions

AG and MV conceived and designed the study. AG conducted the literature research, analysis, and drafted the manuscript with assistance and contributions from MV. MV provided important intellectual content in revising the manuscript. Both authors approved the final manuscript.

### Conflict of Interest Statement

The authors declare that the research was conducted in the absence of any commercial or financial relationships that could be construed as a potential conflict of interest.
