# Supplementary material for: A Systematic Review and Meta-Analysis of Psychological Research on Conspiracy Beliefs: Field Characteristics, Measurement Instruments, and Associations With Personality Traits
Source: Front Psychol. 2019 Feb 11;10:205. doi: 10.3389/fpsyg.2019.00205 (PMC6396711; doi:10.3389/fpsyg.2019.00205)
Supplement: Supplementary file 1 [file Table_1.docx]

Supplementary Material

A systematic review and meta-analysis of psychological research on conspiracy beliefs: Field characteristics, measurement instruments, and associations with personality traits

Andreas Goreis*, Martin Voracek

*** Correspondence:** Andreas Goreis: [andreas.goreis@unive.ac.at](mailto:andreas.goreis@unive.ac.at)

# Supplementary Table

**Supplementary Table 1.** Summary description of the 96 studies used in this review

| Supplementary Table 1 (Continued) | |  |  |  |  |  |  |
| --- | --- | --- | --- | --- | --- | --- | --- |
| Study | *N* | | % Female | Age *M SD* | Variables | Questionnaire | Stimuli/Design |
| Ballová Mikušková (2017) | 119 undergraduates (study 1) 275 undergraduates (study 2) | | 83 82 | 21.33 (1.44) 20.20 (1.48) | Analytic thinking, rational thinking, intelligence (study 1) Media use, analytic thinking, rational thinking (study 2) | GCBS, CMQ (study 1) Slovak Conspiracy Belief Scale (study 2) | – |
| Banas & Miller (2013) | 312 undergraduates | | 53 | 19.99 (1.78) | Perceived threat, counterarguing | 9/11 conspiracy theories | Fact-, logic-, and inoculation-based texts debunking the film clip „Loose Change“, which supports 9/11 conspiracies. Participants watched it afterwards. |
| Barron et al. (2014) | 447 adults | | 77 | 23.17 (7.87) | Schizotypy | BCTI | – |
| Barron et al. (2018) | 411 adults | | 61 | 35.41 (13.06) | Schizotypy, need for cognition, analytic thinking, self-certainty | BCTI | – |
| Bost & Prunier (2013) | 57 undergraduates | | 0 | 19.40 (1.10) | – | 3-item fictional conspiracies | Texts with four fictional conspiracies with high and/or low evidence and/or motive. |
| Bost et al. (2010) | 104 undergraduates | | 0 | – | Religiosity, political affiliation | 4-item fictional conspiracies | Texts with four conspiracies (two well known, two lesser known). |
| Brotherton & Eser (2015) | 150 adults | | 57 | – (14.12) | Boredom proneness, paranoia | GCBS | – |
| Brotherton & French (2014) | 91 adults (study 1) 95 undergraduates (study 2) | | 36 80 | 35.00 (14.30) 21.10 (5.30) | Paranormal belief (study 1) Susceptibility to the conjunction fallacy (study 1 and 2) | CTBS (study 1) GCBS (study 2) | – |
| Brotherton & French (2015) | 83 undergraduates (study 1) 102 undergraduates (study 2) 86 students (study 3) | | – 79 74 | – 21.20 (5.20) 23.50 (9.70) | Anthropomorphism (study 1 and 3) Intentionality (study 2 and 3) | GCBS (study 1, 2, and 3) | – |
| Brotherton et al. (2013) | 489 adults (study 1) 235 undergraduates (study 2) 208 adults (study 3) 194 adults (study 4) | | 46 78 44 45 | 35.90 (13.90) 24.97 (8.76) 40.50 (13.50) 35.00 (14.80) | Interpersonal trust, anomie, paranormal belief, delusions (study 3) Big Five, sensation seeking, emotional intelligence (study 4) | GCBS (study 1, 2, 3, and 4) BCTI (study 2 and 3) 7/7 conspiracies, 9/11 conspiracies, fictitious Red Bull conspiracies (study 2) | – |
| Bruder et al. (2013)^a^ | 7673 adults (study 1a) 133 students (study 1b) 120 students (study 2) 76 adults (study 3) 274 adults (study 4) | | 35 61 50 62 65 | 29.10 (10.20) 24.27 (5.21) – 31.30 (8.10) 25.60 (8.10) | Paranormal belief, paranoia, schizotypy (study 2) Psychosis (unusual experiences), cognitive disorganization, paranoia (study 3) Right-wing authoritarianism, social dominance, control, powerlessness, anomia, death anxiety, anthropomorphism, Big Five (study 4) | CTQ (study 1a, 2, 3, and 4) CMQ (study 1a, 1b, 2, 3, and 4) | – |
| Carey et al. (2016) | 2920 adults | | – | – | US state of birth, favorite NFL team | Specific NFL „deflategate“ conspiracies, 9/11 conspiracies, Obama „birther“ conspiracy | Two texts about group solidarity or elite resentment. |
| Cichocka, Marchlewska, & de Zavala (2016) | 96 students (study 1) 223 students (study 2) 341 MTurk workers (study 3) | | 83 70 45 | 21.64 (1.99) 21.82 (1.77) 31.92 (10.57) | Collective narcissism, collective self-esteem (study 1) Ingroup identification, collective narcissism, perceived threat, anti-Russian attitudes, political affiliation (study 2) Collective narcissism, group-level self-investment (study 3) | Specific anti-Polish conspiracies (study 1) Specific anti-Russian conspiracies (study 2) 11-item GCBS (study 3) | Two conditions: Thinking about ingroup- or outgroup-conspiracies (US government or foreign government, study 3). |
| Cichocka, Marchlewska, de Zavala, & Olechowski (2016) | 202 MTurk workers (study 1) 269 MTurk workers (study 2) | | 37 54 | 31.27 (10.46) 32.81 (12.54) | Self-esteem, narcissism (study 1) Self-esteem, narcissism, collective narcissism, paranoia (study 2) | GCBS (study 1) 11-item GCBS (study 2) | – |
| Dagnall et al. (2015) | 223 adults | | – | 27.79 (12.15) | Schizotypy, delusion, hallucination | GMC, CTQ | – |
| Dagnall et al. (2017) | 246 adults | | 75 | 25.21 (11.51) | Susceptibility to statistical bias, perception of randomness, susceptibility to the conjunction fallacy, paranormal belief | GCBS, GMC | – |
| Darwin et al. (2011) | 120 students | | 50 | – | Paranormal belief, paranoia, schizotypy | CBQ | – |
| Dieguez et al. (2015) | 107 undergraduates (study 1) 123 undergraduates (study 2) 217 adults (study 3) | | 80 83 – | 20.80 (2.10) 21.00 (3.90) 34.60 (9.90) | Randomness perception (study 1, 2, and 3) Political affiliation, pessimism (study 3) | GCBS (study 1, 2, and 3) | Two conditions manipulating the produced sequence of random symbols, they were either „invented“ (neutral intend) or „cheated“ (nefarious intend, study 2 and 3). |
| Douglas & Sutton (2011) | 189 undergraduates (study 1) 60 undergraduates (study 2) | | 86 80 | 20.13 (3.87) 19.83 (2.37) | Machiavellianism (study 1 and 2) PANAS (study 2) | Personal willingness to conspire, 17-item CTBS (study 1 and 2) | Moral prime text (study 2). |
| Douglas et al. (2016) | 202 MTurk workers (study 1) 330 MTurk workers (study 2) | | 50 52 | 32.40 (12.20) 35.45 (13.05) | Anthropomorphism, intentionality (study 1 and 2) Paranormal belief (study 2) | 7-item CTBS (study 1) 17-item CTBS (study 2) | – |
| Drinkwater et al. (2012) | 136 adults | | 50 | 30.58 (14.32) | Paranormal belief, urban legends, reality testing | 10-item specific conspiracies, 5-item generic conspiracies | – |
| Edelson et al. (2017) | 1230 adults | | – | – | Political affiliation, political trust, political interest | Electoral fraud conspiracies | – |
| Einstein & Glick (2015) | 927 MTurk workers | | 47 | 35.10 (–) | Racism, political affiliation, political trust | Conspiracy about faking the unemployment data of the Bureau of Labor Statistics | Exposure to a Bureau of Labor Statistics conspiracy article. |
| Furnham (2013) | 324 adults | | 66 | 23.42 (10.16) | Big Five, religiosity, wealth, political affiliation | 30-item commercial conspiracies | – |
| Galliford et al. (2017) | 323 adults | | 48 | 36.39 (16.17) | Self-esteem, Big Five, religiosity, political affiliation, opinions on the use of alternative medicine | 10-item medical conspiracies, 10-item political conspiracies | – |
| Goertzel(1994) | 348 adults | | – | – | Anomia, interpersonal trust | 10-item specific conspiracies | – |
| Graeupner & Coman (2017) | 119 MTurk workers (study 1) 102 students (study 2) | | 49 65 | 37.23 (13.11) 19.83 (1.85) | PANAS, meaning search (study 1 and 2) Superstitious beliefs (study 2) | 3-item generic conspiracies (study 1 and 2) | Describing unpleasant social event (study 1). Social evaluation/exclusion manipulation (study 2). |
| Green & Douglas (2018) | 246 MTurk workers (study 1) 230 adults (study 2) | | 40 54 | 34.22 (10.07) 38.63 (11.63) | Right-wing authoritarianism, social dominance orientation, trust, Manicheanism (study 1) Attachment style (study 1 and 2) | GCBS (study 1 and 2) 7-item CTBS (study 2) | – |
| Grzesiak-Feldman (2013) | 87 students (study 1) 46 students (study 2) 67 students (study 3) | | 80 0 100 | 20.50 (1.48) 22.42 (1.29) 22.79 (1.94) | Anxiety (study 1, 2, and 3) | 6-item Jewish conspiracies (study 1) 18-item Jewish conspiracies (study 2) 18-item Jewish/German/Arabic conspiracies (study 3) | High-anxiety manipulation 15 min. before an exam (study 2 and 3). |
| Gumhalter (2012)^b^ | 181 adults (study 1) | | 47 | 37.70 (15.90) | Support for democratic principles, Big Five, authoritarianism, right-wing authoritarianism, media exposure, political cynicism, restriction of human rights, religiosity, political affiliation (study 1) | BCTI, 30-item Osama bin Laden conspiracies (study 2) | – |
| Hollander (2017) | 5914 adults | | 52 | 49.44 (16.82) | Media exposure, political affiliation, financial uncertainty, Big Five, interpersonal trust | 4-item specific conspiracies | Representative pre/post 2012 election sample from the American National Election Studies (ANES). |
| Imhoff & Bruder (2014) | 497 MTurk workers (study 1a) 133 students (study 1b) 63 students (study 1c) 294 adults (study 2) 280 adults (study 3) 280 adults (study 4) 1852 adults (study 5) | | 49 62 86 55 59 65 59 | 33.49 (12.25) 24.27 (5.21) 21.90 (4.20) 28.09 (10.41) 23.71 (4.93) 25.63 (8.13) 30.68 (11.97) | Big Five (study 1a)  Right-wing authoritarianism, social dominance orientation (study 1a, 2, 3, 4, and 5) Anti-Semitism, Prejudice against Islam, Antiziganism, Anti-Americanism, anti-capitalism (study 2) Power, likeability, and threat of 32 social groups (study 3 and 4) Perceived control, perceived power, anomia, death anxiety, anthropomorphism, Big Five (study 4) Attributions of responsibility, behavioral intentions, support for nuclear phase-out (study 5) | CMQ (study 1a, 1b, 1c, 2, 3, 4, and 5) | – |
| Imhoff & Lamberty (2017) | 238 MTurk workers (study 1) 465 MTurk workers (study 2) 290 MTurk workers (study 3) | | 48 46 46 | 33.79 (10.39) 34.92 (11.24) 35.74 (10.94) | Need for uniqueness (study 1, 2, and 3) | CMQ (study 1, 2 and 3) 99 specific conspiracies (study 1) 20 specific conspiracies (study 2) „Smoke detectors emanate hyper sound“ conspiracy (study 3) | Text about a „smoke detectors emanating dangerous hyper sound“-conspiracy, either held by the majority or the minority of alleged respondents of a recent survey (study 3). |
| Irwin et al. (2015) | 257 adults | | 77 | 22.10 (7.20) | Doublethink | GCBS, ESCT | – |
| Jolley & Douglas (2014a) | 89 adults (study 1) 188 MTurk workers (parents, study 2) | | 90 60 | 38.06 (9.25) 36.33 (13.40) | Powerlessness concerning vaccination, disillusionment towards those involved in vaccination (study 1 and 2) | 8-item anti-vaccine conspiracies (study 1 and 2) | A scenario in which the participants could decide if they would vaccinate an infant child (study 1 and 2). |
| Jolley & Douglas (2014b) | 168 students (study 1) 191 undergraduates (study 2) | | 64 86 | 22.87 (5.00) 19.75 (3.21) | Trust in institutions, powerlessness towards the government, uncertainty about the government, political engagement (study 1) Powerlessness about climate change, uncertainty about climate change, disillusionment towards climate scientists, trust towards credible sources, climate change behaviors (study 2) | 12-item generic and specific conspiracies (study 1) 7-item climate change conspiracy (study 2) | Anti- or pro-conspiracy texts (study 1 and 2). |
| Jolley & Douglas (2017) | 260 MTurk workers (study 1) 175 adults (study 2) | | 37 54 | 31.90 (9.96) 34.02 (11.10) | Dangers of vaccines (study 1 and 2) | 11-item anti-vaccine conspiracies (study 1 and 2) | Five combinations of arguments in favor or against vaccinations, a scenario in which the participants could decide if they would vaccinate an infant child (study 1 and 2). |
| Jolley et al. (2017) | 98 undergraduates (pilot study) 120 adults (study 1) 159 undergraduates (study 2) 109 adults (study 3) | | 74 57 87 52 | 20.38 (4.38) 34.54 (10.08) 20.00 (5.30) 37.66 (12.32) | Satisfaction with the status quo (pilot study, study 2 and 3) | CTBS, GCBS (pilot study, study 1) Problem in society are cause by „small groups“ (study 3) | System-affirming or system-threat texts (study 1, 2, and 3). Exposure to conspiracy-text (study 2 and 3). |
| Kim & Cao (2016) | 139 students | | 60 | 20.54 (2.75) | Distrust in the government | 4 stories about fictional historic events with conditions weak/strong evidence and good/bad motive | 15-minute film about moon-landing conspiracies. |
| Kumareswaran (2014) | 83 students (study 1) 256 students (study 2) 201 adults (study 4) | | 53 32 60 | 19.55 (6.59) 23.56 (6.65) 27.85 (12.31) | Schizotypy, feeling of control, self-esteem, anomia, authoritarianism, visual pattern perception (study 1 and 2) Fear of death (study 2) Psychopathology, paranoia, delusion, schizotypy, visual pattern perception, powerlessness (study 4) | CBS (study 1 and 4) | High/low feeling of control manipulation (study 1 and 2). |
| Lahrbach & Furnham (2017) | 335 adults | | 66 | 31.60 (13.50) | Modern health worries, trust in doctors, usage of alternative medicine, political affiliation, religiosity | 6-item medical conspiracies | – |
| Lamberty et al. (2018) | 173 adults | | 59 | 31.80 (15.09) | Uncertainty, hindsight, inevitability of the 2016 US election | CMQ | – |
| Lantian et al. (2016) | 152 students (study 1) 267 MTurk workers (study 2) 75 students (study 3) | | 82 60 – | 22.88 (3.53) 34.69 (12.55) – | Paranormal belief, interpersonal trust, self-consciousness (study 3) | OICM (study 1, 2 and 3) GCBS (study 1, 2, and 3) BCTI, CMQ (study 1 and 2) | – |
| Lantian et al. (2017) | 190 adults (study 1) 208 MTurk workers (study 2) 143 students (study 3) | | 62 46 85 | 24.85 (8.53) 32.44 (10.89) 20.93 (4.10) | Perceived scarcity and source of information used to answer the BCTI (study 1) Need for uniqueness (study 2 and 3) | BCTI (study 1) GCBS (study 2) OICM (study 3) | Text about an invented conspiracy, killing several politicians in “Modavia” (study 3). |
| Leiser et al. (2017) | 289 adults | | 65 | 35.24 (12.36) | Big Five, anomia, feelings of control, belief in a dangerous world | BCTI, 14-item specific economic conspiracies | – |
| Lewandowsky et al. (2013) | 1001 adults | | 50 | 43.00 (–) | Opinion about GMOs, climate change, and vaccination, political affiliation | 9-item specific conspiracies | – |
| Lewandowsky et al. (2013) | 1145 adults | | – | – | Free-market ideology, climate change acceptance | 14-item specific conspiracies | – |
| Lobato et al. (2014) | 455 undergraduates | | 65 | 21.00 (5.60) | Need for cognition, desirability of control, Big Five, ontological confusion | Epistemically Unwarranted Beliefs Scale | – |
| Mancosu et al. (2017) | 3027 adults | | – | – | Religiosity, trust in the government, political affiliation, voting intention | 4-item specific conspiracies | – |
| Marchlewska et al. (2017) | 245 adults (study 1) 455 adults (study 2) | | 54 69 | 36.93 (14.21) 30.16 (12.84) | Need for cognitive closure (study 1 and 2) | 6-item conspiracies about refugees and the EU (study 1) 4-item Malaysian Airlines 777 crash conspiracy (study 2) | Text about the EU conducting conspiracies (involving refugees) against Poland (study 1). Text about Malaysian Airline 777 crash conspiracies (study 2). |
| Mashuri & Zaduqisti (2015) | 139 students | | 64 | 19.85 (1.32) | Collective angst | 4-item conspiracies in Indonesia | Text about western countries threatening Islamic countries. |
| Mashuri et al. (2016) | 246 students | | 68 | 21.03 (3.50) | Symbolic threat, realistic threat, identity subversion, dejection-agitation | 5-item conspiracies in Indonesia | – |
| McHoskey (1995) | 253 undergraduates | | 46 | 19.30 (3.10) | Right-wing authoritarianism | 1-item JFK conspiracy | Text about JFK conspiracy. |
| Miller et al. (2016) | 4688 adults | | – | – | Political affiliation, political trust, political knowledge | 4-item specific conspiracies | – |
| Moulding et al. (2016) | 107 adults (study 1) 120 adults (study 2) | | 57 51 | 29.00 (9.50) 28.28 (10.91) | Anomia, alienation (study 1) Uncertainty, need for cognition, alienation, dangerous worldviews, organization of the social world, perception of randomness (study 2) | BCTI, 16-item 9/11 conspiracies, 5-item exposure to 9/11 conspiracies (study 1) BCTI (with false Red Bull conspiracies), GCBS, CMQ (study 2) | – |
| Newheiser et al. (2011) | 144 students (time 1) 50 students (time 2) | | 63 68 | 24.00 (7.54) 23.00 (4.17) | New age beliefs, religiosity, death anxiety, biblical knowledge (time 1 and 2) | 11-item Da Vinci Code conspiracies (time 1 and 2) | Counterevidence to Da Vinci Code conspiracies (time 2). |
| Oliver & Wood (2014) | 1935 adults | | – | – | Political affiliation, supernatural belief, paranormal belief, biblical end times, right-wing authoritarianism, trust | 7-item specific conspiracies | – |
| Oliver & Wood (2014) | 1351 adults | | – | – | Health behaviors | 6-item medical conspiracies | – |
| Orosz et al. (2016) | 813 adults | | 51 | 46.43 (14.74) | Big Five, desirable responding | CAT, CMQ | 4:30 audio about Hungarian conspiracies followed by ridiculing, rational, or empathetic arguments against them. |
| Pasek et al. (2015) | 791 adults | | – | – | Political affiliation, attitudes toward blacks, Obama approval rating | 1-item Obama „birther“ conspiracy | – |
| Pavlova & Silbereisen (2015) | 1212 adults | | 57 | 46.22 (15.82) | Belief in a dangerous world | 5-item H1N1/H1N5-virus conspiracies | – |
| Putra et al. (2015) | 147 students | | 62 | 18.66 (1.86) | Activity in Islamic organization, ingroup essentialism, outgroup essentialism, identity undermining, victim blaming | 3-item Ahmadiyya conspiracies^c^ | – |
| Raab et al. (2013) | 63 adults (study 1) 30 adults (study 2) | | 65 87 | 29.60 (13.30) 22.40 (–) | Self-efficacy (study 1) | 10-item specific conspiracies (study 1) | Card deck with sentences explaining 9/11. Participants chose several cards to construct a narrative (study 2). |
| Radnitz & Underwood (2017) | 1997 adults | | – | – | Self-esteem, authoritarianism, political affiliation | 3-item specific conspiracies | Three texts differing in the situational nature of the conspiracies (anxiety prime or conspirators are identifiable/not identifiable). |
| Richey (2017) | 5860 adults | | – | – | Parenting style, right-wing authoritarianism, trust in the government, political knowledge, political affiliation, life satisfaction | 4-item specific conspiracies | – |
| Rose (2017) | 350 students (study 1a) 305 students (study 1b) 269 students (study 1c) 504 students (study 1d) 665 students (study 2) 1581 adults (study 3) | | 66 61 56 72 63 57 | 19.19 (3.19) 19.61 (3.94) 19.21 (2.32) 19.35 (3.24) 19.41 (3.66) 53.77 (14.41) | Anomia, interpersonal trust, magical thinking (study 1a, 1b, 1c, and 1d) Big Five (study 1a, 1b, and 1c) Aggression, collective self-esteem, interpersonal trust, Big Five, powerlessness, analytic thinking, desirability of control, dogmatism, fantasy proneness, need for cognitive closure, sense of control, anomia, competitive and dangerous worldviews, right-wing authoritarianism, social dominance orientation, anxiety, depression, delusion, paranoia, schizotypy (study 2) Aggression, anomia, anxiety, competitive and dangerous worldview, paranoia, right-wing authoritarianism, social dominance orientation, trust (study 3) | Generalised Conspiracy Belief Scale, Specific Conspiracy Belief Scale (Study 1a, 1b, 1c, 1d, 2, 3) | – |
| Stempel et al. (2007) | 1010 adults | | – | – | Political affiliation, media use, religiosity | 3-item 9/11 conspiracies | – |
| Stieger et al. (2013) | 281 adults | | 60 | 33.83 (15.44) | Self-esteem, paranormal belief, superstitious beliefs, intelligence | 12-item Natascha Kampusch kidnapping conspiracies, BCTI | – |
| Stojanov (2015) | 160 adults | | 70 | 33.39 (–) | Intention to have a fictional child vaccinated, analytic thinking | GCBS | Texts debunking anti-vaccination conspiracies and explaining motives of conspirators. |
| Swami (2012) | 368 students (study 1) 314 adults (study 2) | | 51 52 | 44.15 (12.90) 40.89 (13.04) | Attitudes to authority, political cynicism, political alienation, self-esteem, life satisfaction (study 1) Right-wing authoritarianism, social dominance orientation, attitudes toward Israel, racism (study 2) | BCTI, 12-item Jewish conspiracies (study 1 and 2) | – |
| Swami, Furnham et al. (2016) | 420 MTurk workers | | 54 | 44.68 (12.38) | Stress, stressful life events, state anxiety, trait anxiety, tension | BCTI | – |
| Swami et al. (2010) | 257 adults | | 52 | 43.07 (13.11) | Support for democratic principles, Big Five, authoritarianism, political cynicism | BCTI, 17-item specific 9/11 conspiracies | – |
| Swami et al. (2011) | 817 adults (study 1) 281 adults (study 2) | | 54 60 | 25.18 (9.47) 33.83 (15.44) | Support for democratic principles, political cynicism, attitudes to authority, Big Five, self-esteem, life satisfaction, self-assessed intelligence (study 1) Paranormal belief, superstitious beliefs, intelligence, conformity, self-esteem (study 2) | 12-item 7/7 conspiracies, BCTI (study 1) 12-item fictitious Red-Bull conspiracies, BCTI (study 2) | – |
| Swami & Furnham (2012) | 914 adults | | 47 | 22.40 (4.21) | Big Five, self-esteem, support for democratic principles, political cynicism, self-assessed intelligence | 8-item conspiracies about Amelia Earheart, BCTI | – |
| Swami et al. (2013) | 192 adults (study 1) 392 adults (study 2) | | 50 – | 27.86 (9.45) 32.84 (13.52) | Big Five, extraterrestrial beliefs (study 1) Big Five, extraterrestrial beliefs, schizotypy, new age orientation (study 2) | BCTI, 18-item moon landing conspiracies (study 1 and 2) | Moon landing photographs with or without explaining texts (study 1). |
| Swami et al. (2014) | 990 adults (study 1) 112 undergraduates (study 2) 189 undergraduates (study 3) 140 adults (study 4) | | 57 59 62 47 | 32.79 (13.99) 19.54 (3.06) 19.72 (4.39) 33.87 (15.05) | Analytic thinking, rational thinking, open-minded thinking, need for cognition, need for closure (study 1) One-item Moses Illusion task (study 2 and 4) | BCTI (study 1, 2, and 3) 12-item 7/7 conspiracies (study 4) GCBS (study 4) | Scrambled sentence verbal fluency task (study 2). Hard-to-read font (study 3). |
| Swami, Weis et al. (2016) | 259 MTurk workers | | 50 | 36.36 (11.12) | DSM-5 maladaptive personality traits | BCTI | – |
| Swami, Tran et al. (2016) | 364 adults | | 53 | 32.09 (13.53) | Big Five, anti-scientific attitudes, new age orientation, superstitious beliefs, religiosity | 4-item beliefs in the discovery of the skeleton of a giant | Adopted newspaper article + image. |
| Swami et al. (2017) | 803 MTurk workers | | 56 | 37.07 (11.94) | – | BCTI, CMQ, GCBS, OICM, 10-item specific 9/11 conspiracies | – |
| Swami et al. (2018) | 303 adults | | 59 | 34.73 (12.60) | Brexit referendum voting intentions, clash of civilizations attributions, in-group identification, political knowledge, ambiguity tolerance, Islamophobia, symbolic threat realistic threat | 13-item Islamophobic conspiracies, GCBS | – |
| Uneal (2016) | 355 adults | | 57 | 25.43 (4.12) | Ingroup identification, clash of civilizations, ambiguity intolerance, realistic threat, symbolic threat | 4-item Islamic conspiracies | – |
| Uscinksi et al. (2016) | 1230 adults | | 53 | – | Political affiliation | 4-item generic conspiracies | Cooperative Congressional Election Study of 2012 (CCES). |
| van der Tempel & Alcock (2015) | 246 adults | | 70 | – | Hyperactive agency detection, schizotypy, | GCBS | – |
| van Elk (2015) | 55 adults (study 1) 60 adults (study 2) | | 69 63 | 43.30 (–) 42.40 (–) | Paranormal belief (study 1 and 2) | CBQ (study 2) | Perceptual decision-making tasks (study 1 and 2). |
| van Prooijen & Jostmann (2013) | 73 students (study 1) 91 students (study 2) | | 18 68 | 21.47 (4.75) 20.66 (2.91) | – | 3-item oil companies conspiracies (study 1) 4-item conspiracies about the government of Benin (study 2) | Uncertainty and morality manipulations (study 1 and 2). |
| van Prooijen & van Dijk (2014) | 71 students (study 1) 79 students (study 2) | | 80 51 | 20.82 (–) 21.42 (3.19) | Perspective-taking (study 2) | 3-item conspiracies about the murder of an oppositional leader in Benin (study 1) 4-item conspiracies about the murder of an oppositional leader in Benin (study 2) | Perspective taking and perceived consequences manipulations (study 1 and 2). |
| van Prooijen & Acker (2015) | 119 students (study 1) 1256 adults (study 1) | | 67 38 | 21.31 (3.33) – | Y2K-control consequences, trust in the government (study 2) | 9-item specific conspiracies about city hall corruption involvement in a local metro line construction in Amsterdam (study 1) 5-item specific conspiracies, 1-item Y2K-conspiracy (study 2) | High/low feeling of control manipulation (study 1). |
| van Prooijen (2016) | 84 students (study 1) 81 students (study 2) | | 55 68 | 21.61 (5.03) 20.98 (2.28) | Self-esteem (study 1) Current mood (study 1 and 2) | 10-item specific conspiracies (study 1) 8-item specific conspiracies (study 2) | Belongingness-manipulation (“either imagine yourself in 20 years, happily married or divorced”, study 1 and 2). Uncertainty-manipulation (writing text about situation with great uncertainty, study 2). |
| van Prooijen (2017) | 4062 adults (study 1) 970 adults (study 2) | | 41 47 | 32.25 (12.86) 50.68 (15.85) | Powerlessness, self-esteem, belief in simple solutions (study 1) Feeling of control, analytic thinking, belief in simple solutions (study 2) | 20-item specific conspiracies (study 1) 7-item specific conspiracies (study 2) | – |
| van der Linden (2015) | 316 MTurk workers | | 57 | – | Agreement with climate change, pro-environmental behavior, pro-social intent, political affiliation | – | 2-minute videos about climate change conspiracies. |
| van Prooijen et al. (2015) | 185 MTurk workers (study 1) 1010 adults (study 2a) 1297 adults (study 2b) 268 adults (study 3) | | 52 47 47 26 | – – – – | Political affiliation, paranoia (study 1) Political affiliation, belief in simple solutions (study 2a and 2b) Political affiliation, nonideological attitudes (study 3) | 6-item financial crisis conspiracies, 4-item climate change conspiracies (study 1) 6-item specific conspiracies (study 2a and 2b) 9-item specific conspiracies (study 3) | – |
| Wagner-Egger & Bangerter (2007) | 190 students | | 59 | 22.44 (3.07) | Paranoia, anxiety, anomia, irrationality, simplification of complexity, feeling of control, discrimination against foreign citizens | 8-item specific conspiracies | – |
| Wilson & Rose (2013) | 711 students | | – | – | Paranoia, stress | 25 specific conspiracies | – |
| Wood (2016) | 150 MTurk workers (study 1) 802 MTurk workers (study 2) | | 41 40 | 35.38 (10.94) 32.28 (11.33) | – | GCBS (study 1 and 2) | GCBS items were presented as “conspiracies“ or „ideas“ (study 1). GCBS items were presented as „conspiracies“ or „corruption allegations“(study 2). |
| Wood (2017) | 500 MTurk workers (study 1) 200 MTurk workers (study 2) 200 MTurk workers (study 3) | | 58 46 49 | 35.07 (10.97) 32.68 (9.74) 35.65 (10.52) | Civic participation, pro-environmental behavior (study 3) | FICS (study 1, 2, and 3) 15-item 9/11 conspiracies (study 1, and 2) 8-item vaccine conspiracies (study 2) 5-item US-election conspiracies, 4-item climate change conspiracies (study 3) | – |
| Wood et al. (2012) | 137 undergraduates (study 1) 102 undergraduates (study 2) | | 83 58 | 20.40 (–) 21.00 (–) | – | CTBS (study 1) 3-item Osama bin Laden conspiracies (study 2) | – |

**Supplementary Table 2.** PRISMA 2009 checklist.

| **Section/topic** | **#** | **Checklist item** | **Reported on page #** |
| --- | --- | --- | --- |
| **TITLE** | | |  |
| Title | 1 | Identify the report as a systematic review, meta-analysis, or both. | 1 |
| **ABSTRACT** | | |  |
| Structured summary | 2 | Provide a structured summary including, as applicable: background; objectives; data sources; study eligibility criteria, participants, and interventions; study appraisal and synthesis methods; results; limitations; conclusions and implications of key findings; systematic review registration number. | 2 |
| **INTRODUCTION** | | |  |
| Rationale | 3 | Describe the rationale for the review in the context of what is already known. | 3–5 |
| Objectives | 4 | Provide an explicit statement of questions being addressed with reference to participants, interventions, comparisons, outcomes, and study design (PICOS). | 6–7 |
| **METHODS** | | |  |
| Protocol and registration | 5 | Indicate if a review protocol exists, if and where it can be accessed (e.g., Web address), and, if available, provide registration information including registration number. | NA |
| Eligibility criteria | 6 | Specify study characteristics (e.g., PICOS, length of follow-up) and report characteristics (e.g., years considered, language, publication status) used as criteria for eligibility, giving rationale. | 7 |
| Information sources | 7 | Describe all information sources (e.g., databases with dates of coverage, contact with study authors to identify additional studies) in the search and date last searched. | 7 |
| Search | 8 | Present full electronic search strategy for at least one database, including any limits used, such that it could be repeated. | 7 |
| Study selection | 9 | State the process for selecting studies (i.e., screening, eligibility, included in systematic review, and, if applicable, included in the meta-analysis). | 7 |
| Data collection process | 10 | Describe method of data extraction from reports (e.g., piloted forms, independently, in duplicate) and any processes for obtaining and confirming data from investigators. | 7 |
| Data items | 11 | List and define all variables for which data were sought (e.g., PICOS, funding sources) and any assumptions and simplifications made. | 7–8 |
| Risk of bias in individual studies | 12 | Describe methods used for assessing risk of bias of individual studies (including specification of whether this was done at the study or outcome level), and how this information is to be used in any data synthesis. | NA |
| Summary measures | 13 | State the principal summary measures (e.g., risk ratio, difference in means). | 8 |
| Synthesis of results | 14 | Describe the methods of handling data and combining results of studies, if done, including measures of consistency (e.g., I^2^) for each meta-analysis. | 8 |
| Risk of bias across studies | 15 | Specify any assessment of risk of bias that may affect the cumulative evidence (e.g., publication bias, selective reporting within studies). | NA |
| Additional analyses | 16 | Describe methods of additional analyses (e.g., sensitivity or subgroup analyses, meta-regression), if done, indicating which were pre-specified. | NA |
| **RESULTS** |  |  |  |
| Study selection | 17 | Give numbers of studies screened, assessed for eligibility, and included in the review, with reasons for exclusions at each stage, ideally with a flow diagram. | 7 |
| Study characteristics | 18 | For each study, present characteristics for which data were extracted (e.g., study size, PICOS, follow-up period) and provide the citations. | Supplement Table 1 |
| Risk of bias within studies | 19 | Present data on risk of bias of each study and, if available, any outcome level assessment (see item 12). | NA |
| Results of individual studies | 20 | For all outcomes considered (benefits or harms), present, for each study: (a) simple summary data for each intervention group (b) effect estimates and confidence intervals, ideally with a forest plot. | 8–9, Figures 2–3, Supplement Figures 1–3 |
| Synthesis of results | 21 | Present the main results of the review. If meta-analyses are done, include for each, confidence intervals and measures of consistency. | 14–15 |
| Risk of bias across studies | 22 | Present results of any assessment of risk of bias across studies (see Item 15). | NA |
| Additional analysis | 23 | Give results of additional analyses, if done (e.g., sensitivity or subgroup analyses, meta-regression [see Item 16]). | NA |
| **DISCUSSION** |  |  |  |
| Summary of evidence | 24 | Summarize the main findings including the strength of evidence for each main outcome; consider their relevance to key groups (e.g., healthcare providers, users, and policy makers). | 10 |
| Limitations | 25 | Discuss limitations at study and outcome level (e.g., risk of bias), and at review-level (e.g., incomplete retrieval of identified research, reporting bias). | 11 |
| Conclusions | 26 | Provide a general interpretation of the results in the context of other evidence, and implications for future research. | 11 |
| **FUNDING** |  |  |  |
| Funding | 27 | Describe sources of funding for the systematic review and other support (e.g., supply of data); role of funders for the systematic review. | 11 |

#
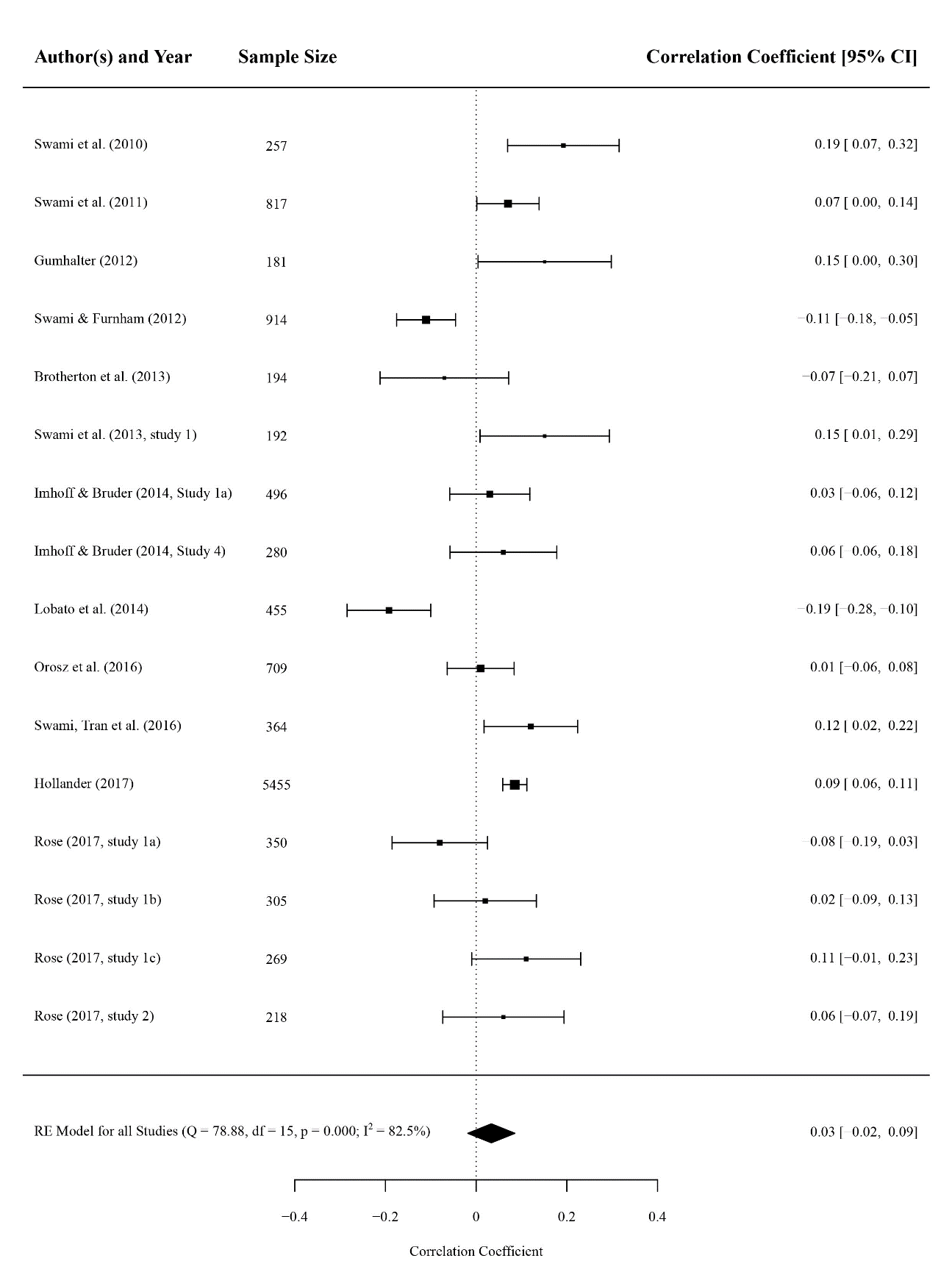
Supplementary Figures

**Supplementary Figure 1.** Forest plot of correlation coefficients between conspiracy beliefs and neuroticism. A positive effect size indicates that higher levels of conspiracy beliefs is associated with higher levels of neuroticism. Average effect was calculated using a random-effects model.


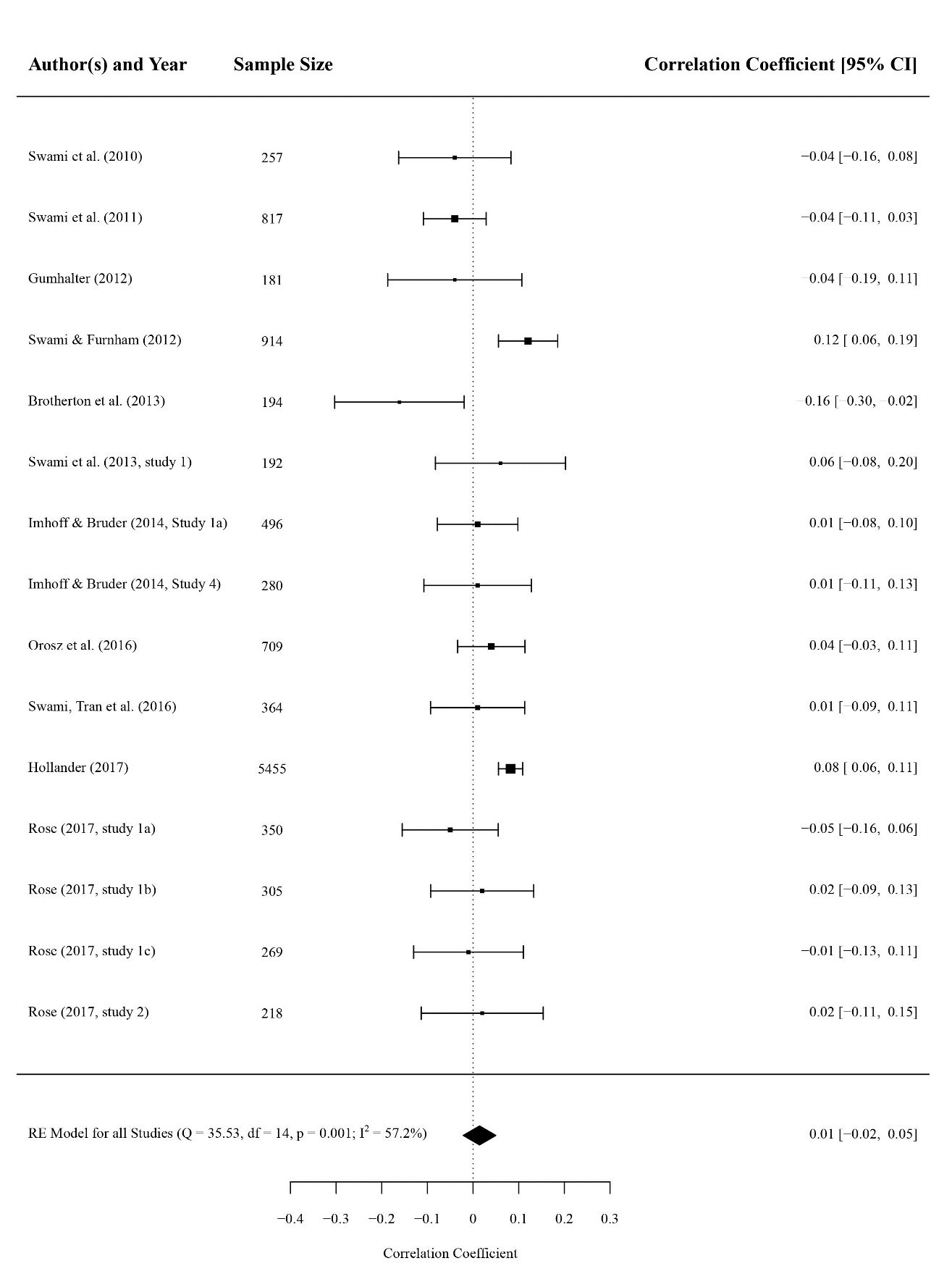


**Supplementary Figure 2.** Forest plot of correlation coefficients between conspiracy beliefs and conscientiousness. A positive effect size indicates that higher levels of conspiracy beliefs is associated with higher levels of conscientiousness. Average effect was calculated using a random-effects model.

**
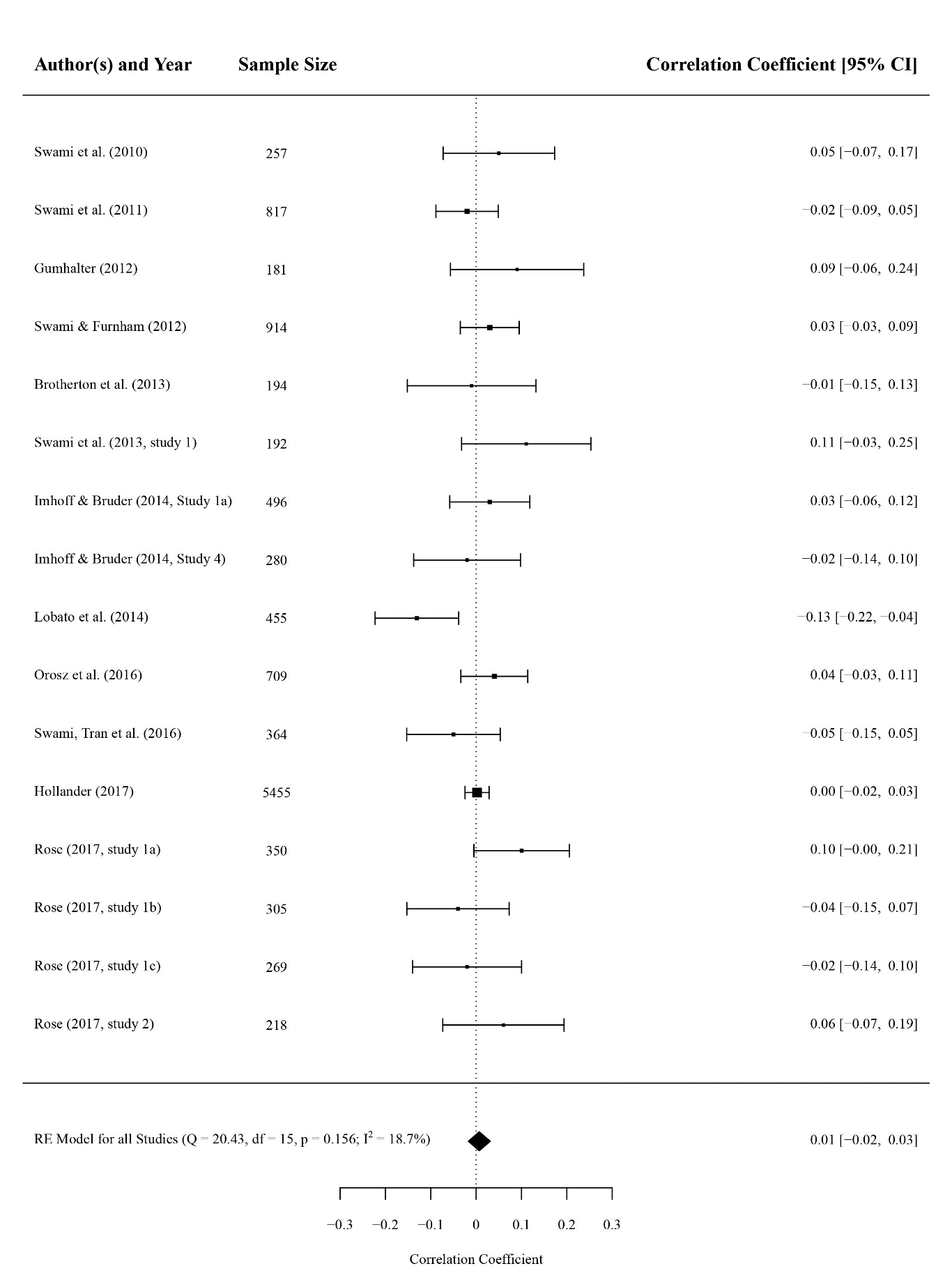
**

**Supplementary Figure 3.** Forest plot of correlation coefficients between conspiracy beliefs and extraversion. A positive effect size indicates that higher levels of conspiracy beliefs is associated with higher levels of extraversion. Average effect was calculated using a random-effects model.

# References

Studies included in the meta-analysis are denoted in the References with an asterisk.

Ballová Mikušková, E. (2017). Conspiracy beliefs of future teachers. *Current Psychology*. Advance online publication. doi:10.1007/s12144-017-9561-4

Banas, J. A., & Miller, G. (2013). Inducing resistance to conspiracy theory propaganda: Testing inoculation and metainoculation strategies. *Human Communication Research*, *39*, 184–207. doi:10.1111/hcre.12000

Barron, D., Furnham, A., Weis, L., Morgan, K. D., Towell, T., & Swami, V. (2018). The relationship between schizotypal facets and conspiracist beliefs via cognitive processes. *Psychiatry Research*, *259*, 15–20. doi:10.1016/j.psychres.2017.10.001

Barron, D., Morgan, K., Towell, T., Altemeyer, B., & Swami, V. (2014). Associations between schizotypy and belief in conspiracist ideation. *Personality and Individual Differences*, *70*, 156–159. doi:10.1016/j.paid.2014.06.040

Bost, P. R., & Prunier, S. G. (2013). Rationality in conspiracy beliefs: The role of perceived motive. *Psychological Reports*, *113*, 118–128. doi:10.2466/17.04.PR0.113x17z0

Bost, P. R., Prunier, S. G., & Piper, A. J. (2010). Relations of familiarity with reasoning strategies in conspiracy beliefs. *Psychological Reports*, *107*, 593–602. doi:10.2466/07.09.17.PR0.107.5.593-602

Brotherton, R., & Eser, S. (2015). Bored to fears: Boredom proneness, paranoia, and conspiracy theories. *Personality and Individual Differences*, *80*, 1–5. doi:10.1016/j.paid.2015.02.011

Brotherton, R., & French, C. C. (2014). Belief in conspiracy theories and susceptibility to the conjunction fallacy. *Applied Cognitive Psychology*, *28*, 238–248. doi:10.1002/acp.2995

Brotherton, R., & French, C. C. (2015). Intention seekers: Conspiracist ideation and biased attributions of intentionality. *PLoS ONE*, *10*, e0124125. doi:10.1371/journal.pone.0124125

*Brotherton, R., French, C. C., & Pickering, A. D. (2013). Measuring belief in conspiracy theories: The generic conspiracist beliefs scale. *Frontiers in Psychology*, *4*, 279. doi:10.3389/fpsyg.2013.00279

Bruder, M., Haffke, P., Neave, N., Nouripanah, N., & Imhoff, R. (2013). Measuring individual differences in generic beliefs in conspiracy theories across cultures: Conspiracy mentality questionnaire. *Frontiers in Psychology*, *4*, 225. doi:10.3389/fpsyg.2013.00225

Carey, J. M., Nyhan, B., Valentino, B., & Liu, M. (2016). An inflated view of the facts? How preferences and predispositions shape conspiracy beliefs about the deflategate scandal. *Research & Politics*, *3*(3), 1–9. doi:10.1177/2053168016668671

Cichocka, A., Marchlewska, M., & de Zavala, A. G. (2016). Does self-love or self-hate predict conspiracy beliefs? Narcissism, self-esteem, and the endorsement of conspiracy theories. *Social Psychological and Personality Science*, *7*, 157–166. doi:10.1177/1948550615616170

Cichocka, A., Marchlewska, M., de Zavala, A. G., & Olechowski, M. (2016). “They will not control us”: Ingroup positivity and belief in intergroup conspiracies. *British Journal of Psychology*, *107*, 556–576. doi:10.1111/bjop.12158

Dagnall, N., Drinkwater, K., Parker, A., Denovan, A., & Parton, M. (2015). Conspiracy theory and cognitive style: A worldview. *Frontiers in Psychology*, *6*, 206. doi:10.3389/fpsyg.2015.00206

Dagnall, N., Denovan, A., Drinkwater, K., Parker, A., & Clough, P. (2017). Statistical bias and endorsement of conspiracy theories. *Applied Cognitive Psychology*, *31*, 368–378. doi:10.1002/acp.3331

Darwin, H., Neave, N., & Holmes, J. (2011). Belief in conspiracy theories. The role of paranormal belief, paranoid ideation and schizotypy. *Personality and Individual Differences*, *50*, 1289–1293. doi:10.1016/j.paid.2011.02.027

Dieguez, S., Wagner-Egger, P., & Gauvrit, N. (2015). Nothing happens by accident, or does it? A low prior for randomness does not explain belief in conspiracy theories. *Psychological Science*, *26*, 1762–1770. doi:10.1177/0956797615598740

Douglas, K. M., & Sutton, R. M. (2011). Does it take one to know one? Endorsement of conspiracy theories is influenced by personal willingness to conspire. *British Journal of Social Psychology*, *50*, 544–552. doi:10.1111/j.2044-8309.2010.02018.x

Douglas, K. M., Sutton, R. M., Callan, M. J., Dawtry, R. J., & Harvey, A. J. (2016). Someone is pulling the strings: Hypersensitive agency detection and belief in conspiracy theories. *Thinking and Reasoning*, *22*, 57–77. doi:10.1080/13546783.2015.1051586

Drinkwater, K., Dagnall, N., & Parker, A. (2012). Reality testing, conspiracy theories and paranormal beliefs. *The Journal of Parapsychology*, *76*, 57–77. Retrieved from https://e-space.mmu.ac.uk/619337/1/dJPSp2012Drinkwater_1.pdf

Edelson, J., Alduncin, A., Krewson, C., Sieja, J. A., & Uscinski, J. E. (2017). The effect of conspiratorial thinking and motivated reasoning on belief in election fraud. *Political Research Quarterly*, *70*, 933–946. doi:10.1177/1065912917721061

Einstein, K. L., & Glick, D. M. (2015). Do I think BLS data are BS? The consequences of conspiracy theories. *Political Behavior*, *37*, 679–701. doi:10.1007/s11109-014-9287-z

Furnham, A. (2013). Commercial conspiracy theories: A pilot study. *Frontiers in Psychology*, *4*, 379. doi:10.3389/fpsyg.2013.00379

Galliford, N., & Furnham, A. (2017). Individual difference factors and beliefs in medical and political conspiracy theories. *Scandinavian Journal of Psychology*, *58*, 422–428. doi:10.1111/sjop.12382

Goertzel, T. (1994). Belief in conspiracy theories. *Political Psychology*, *15*, 731. doi:10.2307/3791630

Graeupner, D., & Coman, A. (2017). The dark side of meaning-making: How social exclusion leads to superstitious thinking. *Journal of Experimental Social Psychology*, *69*, 218–222. doi:10.1016/j.jesp.2016.10.003

Green, R., & Douglas, K. M. (2018). Anxious attachment and belief in conspiracy theories. *Personality and Individual Differences*, *125*, 30–37. doi:10.1016/j.paid.2017.12.023

*Gumhalter, N. (2012). *Dimensionalität und psychologische Korrelate des Glaubens an Verschwörungstheorien* [Dimensionality and psychological correlates of conspiracy belief]. [Master’s thesis]. [Vienna, Austria]: University of Vienna

Grzesiak-Feldman, M. (2013). The effect of high-anxiety situations on conspiracy thinking. *Current Psychology*, *32*, 100–118. doi:10.1007/s12144-013-9165-6

*Hollander, B. A. (2017). Partisanship, individual differences, and news media exposure as predictors of conspiracy beliefs. *Journalism & Mass Communication Quarterly 95,* 691–713. doi:10.1177/1077699017728919

*Imhoff, R., & Bruder, M. (2014). Speaking (Un-)truth to power: Conspiracy mentality as a generalised political attitude. *European Journal of Personality*, *28*, 25–43. doi:10.1002/per.1930

Imhoff, R., & Lamberty, P. K. (2017). Too special to be duped: Need for uniqueness motivates conspiracy beliefs. *European Journal of Social Psychology*, *47*, 724–734. doi:10.1002/ejsp.2265

Irwin, H. J., Dagnall, N., & Drinkwater, K. (2015). Belief inconsistency in conspiracy theorists. *Comprehensive Psychology*, *4*, 19. doi:10.2466/17.CP.4.19

Jolley, D., & Douglas, K. M. (2014a). The effects of anti-vaccine conspiracy theories on vaccination intentions. *PLoS ONE*, *9*, *e89177.* doi:10.1371/journal.pone.0089177

Jolley, D., & Douglas, K. M. (2014b). The social consequences of conspiracism: Exposure to conspiracy theories decreases intentions to engage in politics and to reduce one’s carbon footprint. *British Journal of Psychology*, *105*, 35–56. doi:10.1111/bjop.12018

Jolley, D., & Douglas, K. M. (2017). Prevention is better than cure: Addressing anti-vaccine conspiracy theories. *Journal of Applied Social Psychology*, *47*, 459–469. doi:10.1111/jasp.12453

Jolley, D., Douglas, K. M., & Sutton, R. M. (2017). Blaming a few bad apples to save a threatened barrel: The system-justifying function of conspiracy theories. *Political Psychology*, *39*, 465–478. doi:10.1111/pops.12404

Kim, M., & Cao, X. (2016). The impact of exposure to media messages promoting government conspiracy theories on distrust in the government: Evidence from a two-stage randomized experiment. *International Journal of Communication*, *10*, 3808–3827. Retrieved from http://ijoc.org/index.php/ijoc/article/view/5127/1740

Kumareswaran, D. J. (2014). *The psychopathological foundations of conspiracy theorists.* [Dissertation]. [Wellington, New Zealand]: Victoria University of Wellington

Lahrach, Y., & Furnham, A. (2017). Are modern health worries associated with medical conspiracy theories? *Journal of Psychosomatic Research*, *99*, 89–94. doi:10.1016/j.jpsychores.2017.06.004

Lamberty, P. K., Hellmann, J. H., & Oeberst, A. (2018). The winner knew it all? Conspiracy beliefs and hindsight perspective after the 2016 US general election. *Personality and Individual Differences*, *123*, 236–240. doi:10.1016/j.paid.2017.11.033

Lantian, A., Muller, D., Nurra, C., & Douglas, K. M. (2016). Measuring belief in conspiracy theories: Validation of a French and English single-item scale. *International Review of Social Psychology*, *29*, 1–14. doi:10.5334/irsp.8

Lantian, A., Muller, D., Nurra, C., & Douglas, K. M. (2017). “I know things they don’t know!” the role of need for uniqueness in belief in conspiracy theories. *Social Psychology*, *48*, 160–173. doi:10.1027/1864-9335/a000306

*Leiser, D., Duani, N., & Wagner-Egger, P. (2017). The conspiratorial style in lay economic thinking. *PLoS ONE*, *12,* e0171238. doi:10.1371/journal.pone.0171238

Lewandowsky, S., Gignac, G. E., & Oberauer, K. (2013). The role of conspiracist ideation and worldviews in predicting rejection of science. *PLoS ONE*, *8*, e0075637. doi:10.1371/journal.pone.0075637

Lewandowsky, S., Oberauer, K., & Gignac, G. E. (2013). NASA faked the moon landing—therefore, (climate) science is a hoax: An anatomy of the motivated rejection of science. *Psychological Science*, *24*, 622–633. doi:10.1177/0956797612457686

*Lobato, E., Mendoza, J., Sims, V., & Chin, M. (2014). Examining the relationship between conspiracy theories, paranormal beliefs, and pseudoscience acceptance among a university population. *Applied Cognitive Psychology*, *28*, 617–625. doi:10.1002/acp.3042

Mancosu, M., Vassallo, S., & Vezzoni, C. (2017). Believing in conspiracy theories: Evidence from an exploratory analysis of Italian survey Data. *South European Society and Politics*, *22*, 327–344. doi:10.1080/13608746.2017.1359894

Marchlewska, M., Cichocka, A., & Kossowska, M. (2017). Addicted to answers: Need for cognitive closure and the endorsement of conspiracy beliefs. *European Journal of Social Psychology, 48*, 109–117. doi:10.1002/ejsp.2308

Mashuri, A., & Zaduqisti, E. (2015). The effect of intergroup threat and social identity salience on the belief in conspiracy theories over terrorism in Indonesia: Collective angst as a mediator. *International Journal of Psychological Research*, *8*, 24–35. doi:10.21500/20112084.642

Mashuri, A., Zaduqisti, E., Sukmawati, F., Sakdiah, H., & Suharini, N. (2016). The role of identity subversion in structuring the effects of intergroup threats and negative emotions on belief in anti-west conspiracy theories in Indonesia. *Psychology and Developing Societies*, *28*, 1–28. doi:10.1177/0971333615622893

McHoskey, J. W. (1995). Case closed? On the John F. Kennedy assassination: Biased assimilation of evidence and attitude polarization. *Basic and Applied Social Psychology*, *17*, 395–409. doi:10.1207/s15324834basp1703_7

Miller, J. M., Saunders, K. L., & Farhart, C. E. (2016). Conspiracy endorsement as motivated reasoning: The moderating roles of political knowledge and trust. *American Journal of Political Science*, *60*, 824–844. doi:10.1111/ajps.12234

Moulding, R., Nix-Carnell, S., Schnabel, A., Nedeljkovic, M., Burnside, E. E., Lentini, A. F., & Mehzabin, N. (2016). Better the devil you know than a world you don’t? Intolerance of uncertainty and worldview explanations for belief in conspiracy theories. *Personality and Individual Differences*, *98*, 345–354. doi:10.1016/j.paid.2016.04.060

Newheiser, A. K., Farias, M., & Tausch, N. (2011). The functional nature of conspiracy beliefs: Examining the underpinnings of belief in the Da Vinci code conspiracy. *Personality and Individual Differences*, *51*, 1007–1011. doi:10.1016/j.paid.2011.08.011

Oliver, J. E., & Wood, T. (2014). Medical conspiracy theories and health behaviors in the United States. *JAMA Internal Medicine*, *174*, 817–818. doi:10.1001/jamainternmed.2014.190

Oliver, J. E., & Wood, T. J. (2014). Conspiracy theories and the paranoid style(s) of mass opinion. *American Journal of Political Science*, *58*, 952–966. doi:10.1111/ajps.12084

*Orosz, G., Krekó, P., Paskuj, B., Tóth-Király, I., Bothe, B., & Roland-Lévy, C. (2016). Changing conspiracy beliefs through rationality and ridiculing. *Frontiers in Psychology*, *7*, 1525. doi:10.3389/fpsyg.2016.01525

Pasek, J., Stark, T. H., Krosnick, J. A., & Tompson, T. (2015). What motivates a conspiracy theory? Birther beliefs, partisanship, liberal-conservative ideology, and anti-Black attitudes. *Electoral Studies*, *40*, 482–489. doi:10.1016/j.electstud.2014.09.009

Pavlova, M. K., & Silbereisen, R. K. (2015). Supportive social context and intentions for civic and political participation: An application of the theory of planned behaviour. *Journal of Community & Applied Social Psychology*, *25*, 432–446. doi:10.1002/casp

Putra, I. E., Mashuri, A., & Zaduqisti, E. (2015). Demonising the victim: Seeking the answer for how a group as the violent victim is blamed. *Psychology and Developing Societies*, *27*, 31–57. doi:10.1177/0971333614564741

Raab, M. H., Ortlieb, S. A., Auer, N., Guthmann, K., & Carbon, C. C. (2013). Thirty shades of truth: Conspiracy theories as stories of individuation, not of pathological delusion. *Frontiers in Psychology*, *4*, 406. doi:10.3389/fpsyg.2013.00406

Radnitz, S., & Underwood, P. (2017). Is belief in conspiracy theories pathological? A survey experiment on the cognitive roots of extreme suspicion. *British Journal of Political Science*, *47*, 113–129. doi:10.1017/S0007123414000556

Richey, S. (2017). A Birther and a truther: The influence of the authoritarian personality on conspiracy beliefs. *Politics and Policy*, *45*, 465–485. doi:10.1111/polp.12206

*Rose, C. L. (2017). *The measurement and prediction of conspiracy beliefs.* [Dissertation]. [Wellington, New Zealand]: Victoria University of Wellington

Stempel, C., Hargrove, T., & Stempel, G. H. (2007). Media use, social structure, and belief in 9/11 conspiracy theories. *Journalism and Mass Communication Quarterly*, *84*, 353–372. doi:10.1177/107769900708400210

Stieger, S., Gumhalter, N., Tran, U. S., Voracek, M., & Swami, V. (2013). Girl in the cellar: A repeated cross-sectional investigation of belief in conspiracy theories about the kidnapping of Natascha Kampusch. *Frontiers in Psychology*, *4*, 297. doi:10.3389/fpsyg.2013.00297

Stojanov, A. (2015). Reducing conspiracy theory beliefs. *Psihologija*, *48*, 251–266. doi:10.2298/PSI1503251S

Swami, V. (2012). Social psychological origins of conspiracy theories: The case of the Jewish conspiracy theory in Malaysia. *Frontiers in Psychology*, *3*, 280. doi:10.3389/fpsyg.2012.00280

Swami, V., Furnham, A., Smyth, N., Weis, L., Lay, A., & Clow, A. (2016). Putting the stress on conspiracy theories: Examining associations between psychological stress, anxiety, and belief in conspiracy theories. *Personality and Individual Differences*, *99*, 72–76. doi:10.1016/j.paid.2016.04.084

*Swami, V., Chamorro-Premuzic, T., & Furnham, A. (2010). Unanswered questions: A preliminary investigation of personality and individual difference predictors of 9/11 conspiracist beliefs. *Applied Cognitive Psychology*, *24*, 749–761. doi:10.1002/acp.1583

*Swami, V., Coles, R., Stieger, S., Pietschnig, J., Furnham, A., Rehim, S., & Voracek, M. (2011). Conspiracist ideation in Britain and Austria: Evidence of a monological belief system and associations between individual psychological differences and real-world and fictitious conspiracy theories. *British Journal of Psychology*, *102*, 443–463. doi:10.1111/j.2044-8295.2010.02004.x

*Swami, V., & Furnham, A. (2012). Examining conspiracist beliefs about the disappearance of Amelia Earhart. *Journal of General Psychology*, *139*, 244–259. doi:10.1080/00221309.2012.697932

*Swami, V., Pietschnig, J., Tran, U. S., Nader, I. W., Stieger, S., & Voracek, M. (2013). Lunar lies: The impact of informational framing and individual differences in shaping conspiracist beliefs about the moon landings. *Applied Cognitive Psychology*, *27*, 71–80. doi:10.1002/acp.2873

Swami, V., Voracek, M., Stieger, S., Tran, U. S., & Furnham, A. (2014). Analytic thinking reduces belief in conspiracy theories. *Cognition*, *133*, 572–585. doi:10.1016/j.cognition.2014.08.006

Swami, V., Weis, L., Lay, A., Barron, D., & Furnham, A. (2016). Associations between belief in conspiracy theories and the maladaptive personality traits of the personality inventory for DSM-5. *Psychiatry Research*, *236*, 86–90. doi:10.1016/j.psychres.2015.12.027

*Swami, V., Tran, U. S., Stieger, S., Pietschnig, J., Nader, I. W., & Voracek, M. (2016). Who believes in the giant skeleton myth? An examination of individual difference correlates. SAGE Open, 6. doi:10.1177/2158244015623592

Swami, V., Barron, D., Weis, L., Voracek, M., Stieger, S., & Furnham, A. (2017). An examination of the factorial and convergent validity of four measures of conspiracist ideation, with recommendations for researchers. *PLoS ONE*, *12*, 1–27. doi:10.1371/journal.pone.0172617

Swami, V., Barron, D., Weis, L., & Furnham, A. (2018). To Brexit or not to Brexit: The roles of Islamophobia, conspiracist beliefs, and integrated threat in voting intentions for the United Kingdom European Union membership referendum. *British Journal of Psychology*, *109*, 156–179. doi:10.1111/bjop.12252

Uenal, F. (2016). The “Secret Islamization” of Europe: Exploring integrated threat theory for predicting Islamophobic conspiracy stereotypes. *International Journal of Conflict and Violence*, *10*, 93–108. doi:10.41119/UNIBI/ijcv.499

Uscinski, J. E., Klofstad, C., & Atkinson, M. D. (2016). What drives conspiratorial beliefs? The role of informational cues and predispositions. *Political Research Quarterly*, *69*, 57–71. doi:10.1177/1065912915621621

van der Tempel, J., & Alcock, J. E. (2015). Relationships between conspiracy mentality, hyperactive agency detection, and schizotypy: Supernatural forces at work? *Personality and Individual Differences*, *82*, 136–141. doi:10.1016/j.paid.2015.03.010

van Elk, M. (2015). Perceptual biases in relation to paranormal and conspiracy beliefs. *PLoS ONE*, *10*, e0130422. doi:10.1371/journal.pone.0130422

van Prooijen, J. W. (2016). Sometimes inclusion breeds suspicion: Self-uncertainty and belongingness predict belief in conspiracy theories. *European Journal of Social Psychology*, *46*, 267–279. doi:10.1002/ejsp.2157

van Prooijen, J. W. (2017). Why education predicts decreased belief in conspiracy theories. *Applied Cognitive Psychology*, *31*, 50–58. doi:10.1002/acp.3301

van Prooijen, J. W., & Acker, M. (2015). The influence of control on belief in conspiracy theories: Conceptual and applied extensions. *Applied Cognitive Psychology*, *29*, 753–761. doi:10.1002/acp.3161

van Prooijen, J. W., & Jostmann, N. B. (2013). Belief in conspiracy theories: The influence of uncertainty and perceived morality. *European Journal of Social Psychology*, *43*, 109–115. doi:10.1002/ejsp.1922

van Prooijen, J. W., & van Dijk, E. (2014). When consequence size predicts belief in conspiracy theories: The moderating role of perspective taking. *Journal of Experimental Social Psychology*, *55*, 63–73. doi:10.1016/j.jesp.2014.06.006

van Prooijen, J. W., Krouwel, A. P. M., & Pollet, T. V. (2015). Political extremism predicts belief in conspiracy theories. *Social Psychological and Personality Science*, *6*, 570–578. doi:10.1177/1948550614567356

van der Linden, S. (2015). The conspiracy-effect: Exposure to conspiracy theories (about global warming) decreases pro-social behavior and science acceptance. *Personality and Individual Differences*, *87*, 171–173. doi:10.1016/j.paid.2015.07.045

Wagner-Egger, P., & Bangerter, A. (2007). La vérité est ailleurs: Corrélats de l’adhésion aux théories du complot [The truth lies elsewhere: Correlates adherence to conspiracy belief]. *Revue Internationale de Psychologie Sociale,* *20*(4), 31–61. Retrieved from https://www.cairn.info/revue-internationale-de-psychologie-sociale-2007-4-page-31.html

Wilson, M. S., & Rose, C. (2013). The role of paranoia in a dual-process motivational model of conspiracy belief. In J. W. van Prooijen & P. A. M. van Lange (Eds.), *Power, Politics, and Paranoia* (pp. 273–291). Cambridge: Cambridge University Press. doi:10.1017/CBO9781139565417.019

Wood, M. J. (2016). Some dare call it conspiracy: Labeling something a conspiracy theory does not reduce belief in it. *Political Psychology*, *37*, 695–705. doi:10.1111/pops.12285

Wood, M. J. (2017). Conspiracy suspicions as a proxy for beliefs in conspiracy theories: Implications for theory and measurement. *British Journal of Psychology*, *108*, 507–527. doi:10.1111/bjop.12231

Wood, M. J., Douglas, K. M., & Sutton, R. M. (2012). Dead and alive: Beliefs in contradictory conspiracy theories. *Social Psychological and Personality Science*, *3*, 767–773. doi:10.1177/1948550611434786
